# Lightweight and compact smart walking cane

**DOI:** 10.7717/peerj-cs.1563

**Published:** 2023-11-29

**Authors:** Gonçalo Neves, João S. Sequeira, Cristina P. Santos

**Affiliations:** 1 Instituto Superior Técnico, Universidade de Lisboa, Lisbon, Portugal; 2 Department of Industrial Electronics, Universidade do Minho, Minho, Portugal

**Keywords:** Robot cane, Locomotion assistance, Mild locomotion disabilities, Inertial unit, Full-state feedback, Unicycle kinematics, Lightweight and compact smart cane

## Abstract

Devices such as canes and wheelchairs, used to assist locomotion, have remained mostly unchanged for centuries. Recent advances in robotics have the potential to develop smart versions of these devices that can offer better support and living conditions to their users. This is the underlying objective of this project. The existing devices, used during recovery and rehabilitation phases where gait stability is key, are often bulky and cannot be easily migrated from hospital to domestic environments, where maneuvering space tends to be restricted. This article discusses a compact, lightweight and minimally invasive, robotic cane to assist locomotion. The device can assist users with mild locomotion disabilities, *e.g.*, in the final stages of rehabilitation, to maintain and recover their balance in standing and walking situations. This extends previous experiments with alternative control strategies, merged with indicators (based on the Gini index) able to recognize differences between users. Several experiments, with a range of users possessing different mobility impairments, confirm the viability of the robotic cane, with users comfortably using the cane after three minutes, on average, proving its ease of use and low intrusiveness, and with constant support offered during the whole movement. Furthermore, the real-time tuning of the controller gains, *via* the Gini inequality index, enables adjustment to the user’s movement.

## Introduction

Every year, the number of people with some type of disability increases due to a rise in chronic health conditions and an ageing population, accounting for 15% of the world’s population in 2021. Mobility impairments represent a large portion of this, with about 1% of people worldwide having rheumatoid arthritis and 1.85% requiring a wheelchair. This loss of mobility leads to a deterioration of not only the physical health of the individual but also their psychological, social and economic welfare (Winter, 2009). Also, the constant increase of the elderly population that has been observed in recent years is projected to continue, doubling today’s numbers by 2050 (United Nations Department of Economic and Social Affairs, Population Division, 2020), this being the group that tends to suffer the most from movement restrictions, with many of them associated with muscle weakness and fatigue and/or other pathologies that usually come with age. Coupling these two factors, the number of people that suffer from mobility loss is becoming increasingly significant.

With the current advancements in robotics, devices that aid walking by operating on the regeneration of the walking ability of patients, and on reducing the tasks and stress of healthcare professionals derived from the constant supervision that this type of user requires, are becoming not only feasible but increasingly more sophisticated.

Most of the studies carried out focus on Smart Walkers (SWs), robust and large devices, but generally offering great support and a wide range of sensors to intensively monitor the user. This results in a more intrusive application with third-party intervention, reducing the possibility of its use outside of clinical and hospital environments, namely in domestic environments (Moreira et al., 2019; Ohnuma, Lee & Chong, 2011; Rentschler et al., 2008; Neto et al., 2015; Grondin & Li, 2013; Jun et al., 2011; Schraft, Schaeffer & May, 1998).

Some of these problems can be overcome by devices such as Smart Canes (SCs), which are generally light and more compact, making them viable for unattended home use. This type of device can also be used to collect information on how the cane is used, which can then be communicated to the medical team for further analysis or real-time warnings, creating a complete and secure monitoring.

Some of the work already carried out on SCs is presented in Naeem & Assal (2019), Shim & Yoon (2003), Spenko, Yu & Dubowsky (2006), Wang et al. (2015), Van Lam & Fujimoto (2017), Stramel et al. (2019), Trujillo-León et al. (2020) and Ady, Bachta & Bidaud (2014).

The present article extends the project presented at the 9th International Conference on Robot Intelligence Technology and Applications (RiTA 2021), (Neves, Sequeira & Santos, 2022), with the addition of new tests performed with different types of surfaces and different control strategies. While the initial testing clearly pointed to the validity of the concept, it also highlighted challenges such as adjusting the behaviour of the cane to the user specific gait features and the need to consider surfaces with different characteristics, as in indoors and outdoors scenarios. Using inequality indexes to adjust the controller (and hence the behaviour) of the cane is a novel pathway proposed in the article.

This robotic cane is a less intrusive, highly mobile and versatile solution. The control uses a model similar to the classic inverted pendulum problem. Its behaviour is predictable and stable, making the device suitable for individuals who still have some control over their locomotion but need support. This is the case of users in the second stage of recovery from diseases that affect gait, after a more severe first phase of rehabilitation in a clinical environment with walkers or other robust devices, but can now continue treatment in a domestic environment with the aid of a more compact and less intrusive device.

Most of the SCs in the literature are still too big to provide a comfortable usage in tight/cluttered places and/or require sensors to be physically connected to the user. The device presented in this article is compact and lightweight, and does not require sensors attached to the user. These are important characteristics in maintaining a low level of intrusiveness. Experimental results show that the system is easy to use—with the users quickly adapting themselves to the device—and tune.

This article has the following structure. ‘Materials and Methods’ gives details about aspects of its implementation. ‘Results and Discussion’ presents tests with real users. ‘Conclusions’ points to conclusions and future work.

## Materials and Methods

The main feature that differentiates this device from others in the literature is its reduced volume and high mobility. The system is formed by a single wheel connected to a standard cane, not only minimizing its footprint but also the weight and mechanical complexity of the device. It allows the user to move freely, leaving enough space to move the lower limbs, and allows a very predictable movement due to the simplicity of the system.

As the single wheel only allows longitudinal movement control, the lateral movement has to be manually controlled by the user, similarly to a traditional cane. Current tests have shown that lateral movement is rarely necessary and users quickly learn that by slightly rotating the cane they can make small lateral displacements. Also, the lateral stability, intrinsic to the configuration used, was found to be important. A design with omnidirectional movement has additional stability issues and is less predictable, and so was not considered at this stage.

It is assumed that the wheel does not slip in either direction, allowing a simplification of the robot’s behaviour and making it more predictable for the user.

### Model of the robotic cane

**Figure 1:**
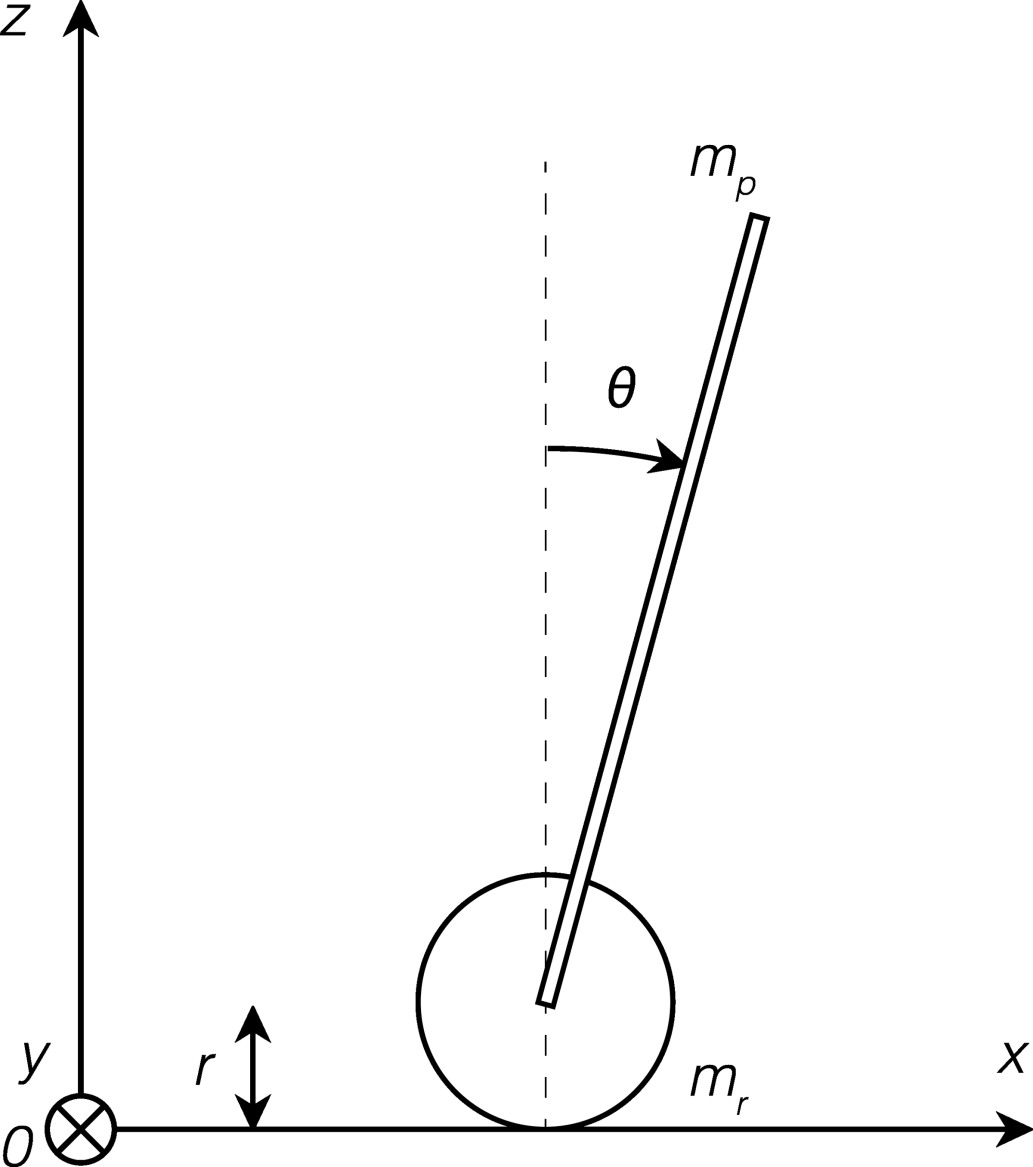
Schematic of the model.

The proposed cane is similar to an inverted pendulum mounted on a cart, with only one controllable degree of freedom, namely, the traction wheel. Figure 1 shows a simplified schematic of the model of the cane. Details about the variables of the model as well as their respective values are given in Tables 1 and 2, respectively.

The linear space state model of the cane was derived based on the forces applied perpendicularly to the handle and horizontally to the wheel, as in Fig. 1. After being converted to matrix form, it results in the system represented in Eq. (1).

(1) \begin{eqnarray*} \left[ \begin{array}{@{}c@{}} \displaystyle \dot {x}\\ \displaystyle \ddot{x}\\ \displaystyle \dot {\theta }\\ \displaystyle \ddot{\theta } \end{array} \right] = \left[ \begin{array}{@{}c@{}} \displaystyle 0~1~0~0\\ \displaystyle 0~0~0~- \frac{{m}_{p}l}{{m}_{r}+{m}_{p}} \\ \displaystyle 0~0~0~1\\ \displaystyle 0~- \frac{ \frac{{m}_{r}r}{2} }{ \frac{1}{3} {m}_{p}{l}^{2}} ~- \frac{ \frac{{m}_{p}gl}{2} }{ \frac{1}{3} {m}_{p}{l}^{2}} ~- \frac{\delta }{ \frac{1}{3} {m}_{p}{l}^{2}} \end{array} \right] \left[ \begin{array}{@{}c@{}} \displaystyle x\\ \displaystyle \dot {x}\\ \displaystyle \theta \\ \displaystyle \dot {\theta } \end{array} \right] + \left[ \begin{array}{@{}c@{}} \displaystyle 0\\ \displaystyle \frac{ \frac{1}{r} }{{m}_{r}+{m}_{p}} \\ \displaystyle 0\\ \displaystyle \frac{1}{ \frac{1}{3} {m}_{p}{l}^{2}} \end{array} \right] u\text{,}y= \left[ \begin{array}{@{}c@{}} \displaystyle 1~0~0~0\\ \displaystyle 0~0~1~0 \end{array} \right] \left[ \begin{array}{@{}c@{}} \displaystyle x\\ \displaystyle \dot {x}\\ \displaystyle \theta \\ \displaystyle \dot {\theta } \end{array} \right] + \left[ \begin{array}{@{}c@{}} \displaystyle 0\\ \displaystyle 0 \end{array} \right] u\text{.}\end{eqnarray*}

**Figure 2:**
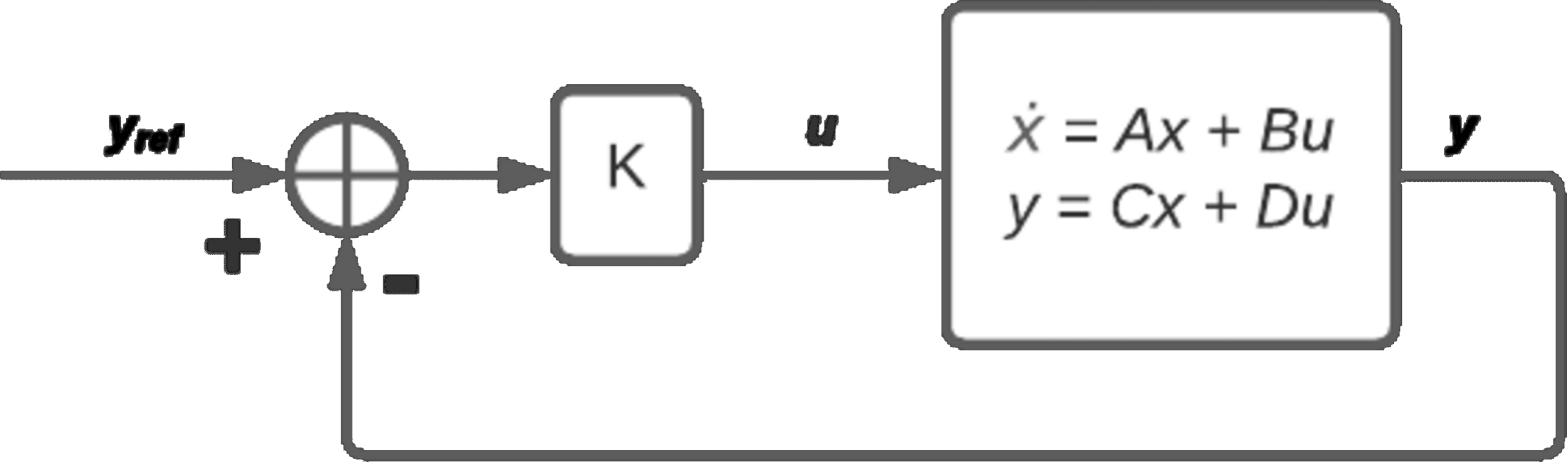
Control diagram.

It can be shown that the vertical angle is observable and controllable. Its control is done through a full-state feedback control (Eq. (2)), where the gain *K* is obtained with a linear quadratic regulator (LQR) by minimizing the quadratic cost function Eq. (3) (Lemos, 2019), as shown in Fig. 2. The state and control weights are set to *Q* = *C*^*T*^*C* and *R* = 0.0001, to improve the response to variations in the user’s input.

**Table 1:** Mathematical model variables.

*θ* (rad)	angle of the rod
*x* (m)	position of the wheel
*m*_*p*_ (kg)	rod mass
*m*_*r*_ (kg)	wheel mass
*g* (m/s^2^)	acceleration due to gravity
*r* (m)	radius of the wheel
*l* (m)	length of the rod
*l*_*c*_ (m)	length from axle to rod’s centre of mass
*δ* (N m s/rad)	viscous friction coefficient
*u* (V)	control input (voltage supplied to motors)

**Table 2:** Parameters for the smart cane.

Mass of the rod (kg)	1.330
Mass of the wheel (kg)	0.080
Radius of the wheel radius (m)	0.073
Length of the rod (middle position) (m)	0.800
Length from axle to rod’s centre of mass (m)	0.147
Viscous friction coefficient (N m s/rad)	0.25

(2) \begin{eqnarray*}u& =-KX\end{eqnarray*}

(3) \begin{eqnarray*}J(u)& =\int \nolimits \nolimits _{0}^{\infty }({x}^{\top }Qx+{u}^{\top }Ru)dt\end{eqnarray*}

### Prototype of the smart cane

The prototype in this article is the one in Neves, Sequeira & Santos (2022), an upgraded version of the one presented in Neves, Sequeira & Santos (2021), including a stronger motor. The users in the trials of this cane provided written informed consent, and the study was approved by the ACES (Agrupamento de Centros de Saúde) Loures-Odivelas. All tests were performed under the supervision of a medical team.

The device is controlled with an Arduino Uno. It receives data from the inertial measurement unit (IMU) and calculates the control signal that is sent to the worm gear traction motor (through a motor driver). Force sensitive resistors (FSR) on the handle measure the force applied by the user to the device. Electric energy is available from two lithium polymer (LiPo) batteries, to separate the power circuits of the motor and the micro-controller. The sensor data acquired is sent *via* a Bluetooth module to a computer for storage and further analysis. The details of all the components of this device and their connection diagrams can be found in Neves (2021).

The FSR used has a limit of 10 N, and is calibrated at the start of each trial to compensate for the grasping force applied by the user, hence most measurements of the force appear to be limited to 6 N.

Accelerometer and gyro data from the IMU are processed and combined using a Kalman filter, with process noise variance for the accelerometer and the gyro bias set to *Q*_*angle*_ = 0.001 and *Q*_*bias*_ = 0.003, respectively, and measurement noise variance set to *R*_*measure*_ = 0.03.

**Figure 3:**
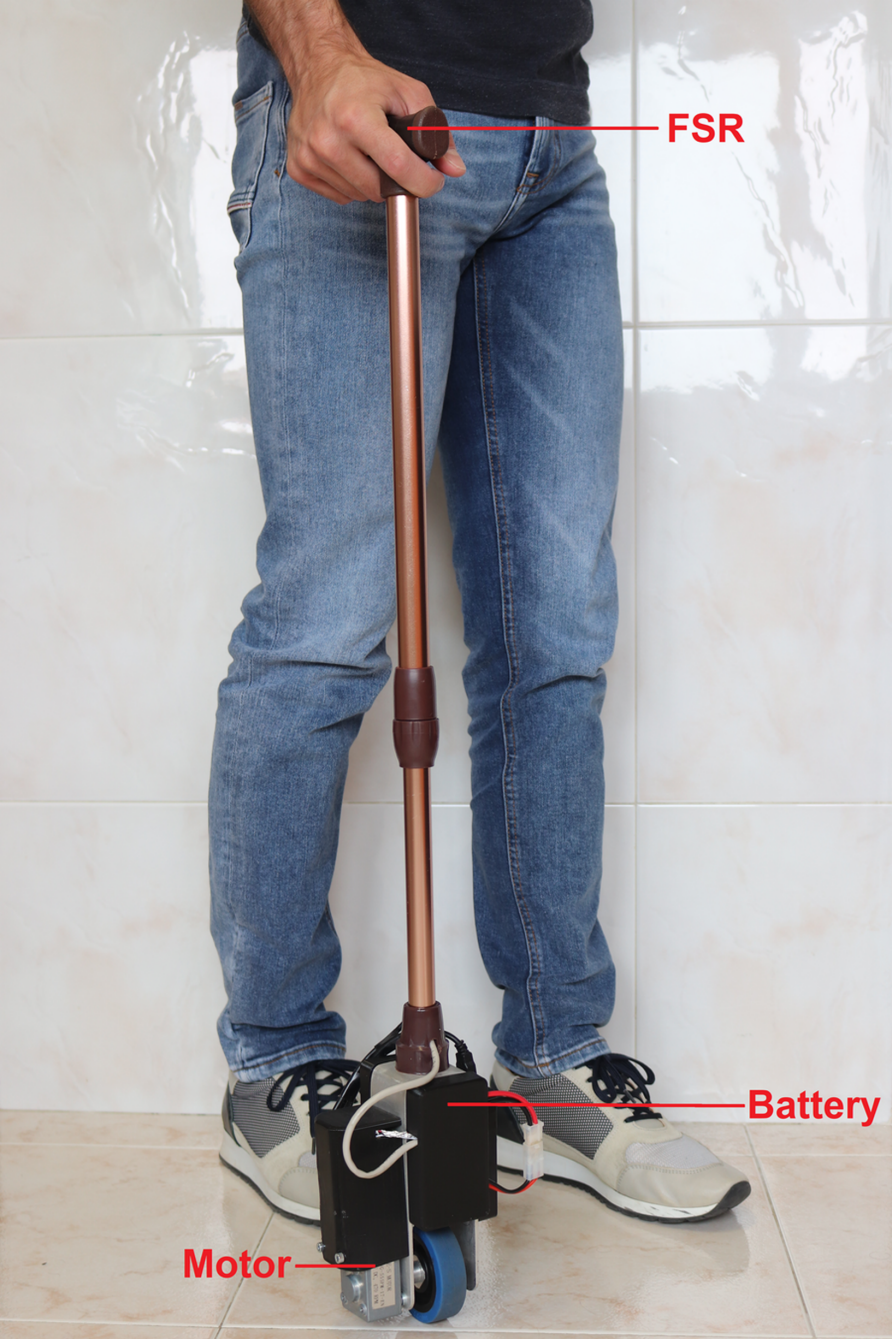
Prototype of the smart cane.

To reduce the weight while maintaining structural strength, the whole frame of the cane is aluminium (Fig. 3), allowing the complete prototype to weigh under 1.410 kg, significantly less than the 16.7 kg average mass a person with 60 years or older can lift from the hip to the shoulder (in four consecutive repetitions) (Matheson et al., 2014). The heaviest components (batteries and motor) are placed near the wheel, lowering the centre of mass. The battery lasts approximately two hours in continuous operation.

The physical parameters of the robotic cane are detailed in Table 2.

Due to the small and mostly unconscious variations induced by the user’s hand, *e.g.*, at old ages where problems such as Parkinson’s are common, a ±5^∘^ gap was placed around the vertical position where the control signal is reduced by 80% to prevent small excessive variations that could compromise the comfort and safety of the cane.

Conversations with end users and healthcare technicians led to the conclusion that the optimal position of the cane to offer comfortable support to the user was not at 0^∘^, but when the cane was approximately −7^∘^, inclined towards the user, so this was set as the reference angle of the cane, and will hereafter be referred to as the vertical position of the cane.

### Control methods

The adjustment of the motion of the cane to the user gait relies on three alternatives. The first method amounts to a common LQR controller, described in Neves, Sequeira & Santos (2021), Neves, Sequeira & Santos (2022).

**Figure 4:**
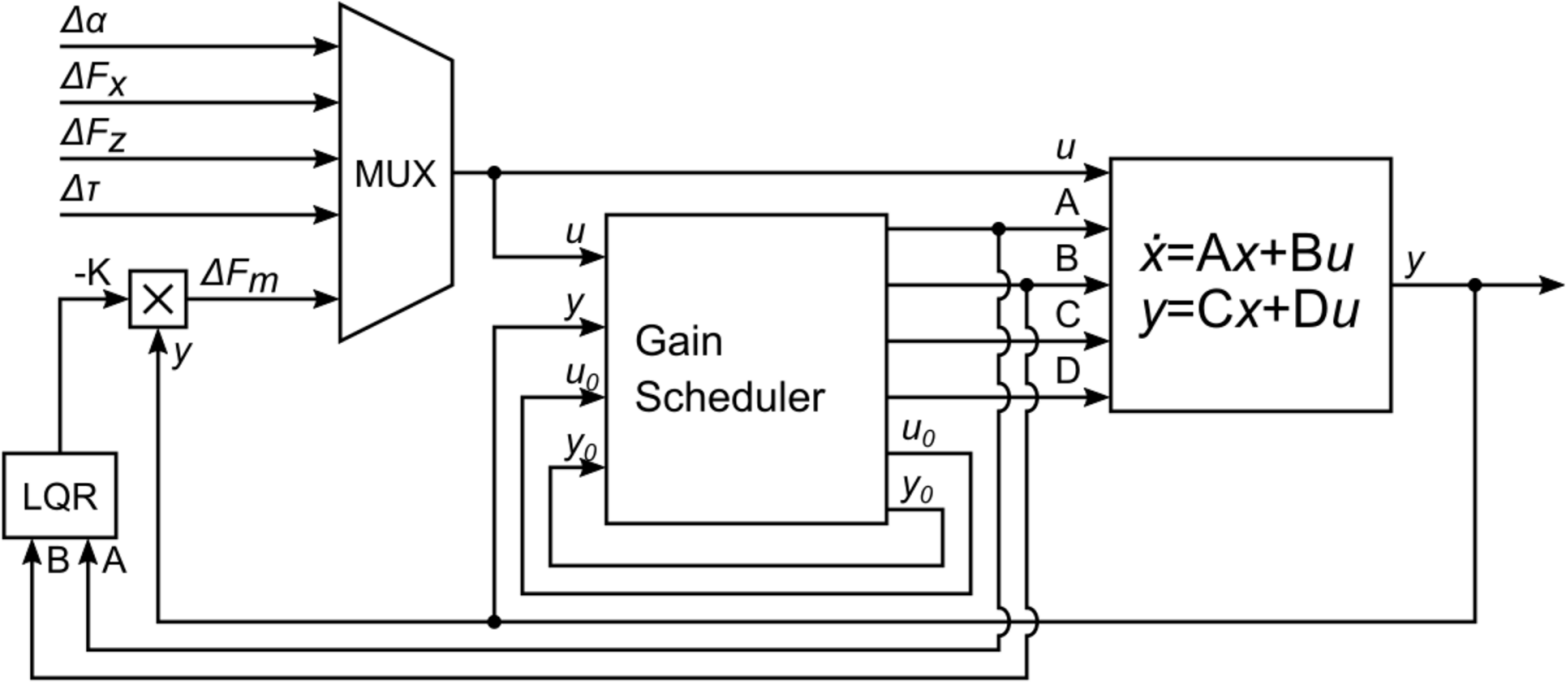
Gain-scheduled LQR feedback controller.

A second method adds a gain scheduling procedure to the LQR controller is detailed in Bollinger, Neves & Sequeira (2022). As with the previous method, this control method uses LQR, but gain scheduling is used to choose, at each moment, the linearized model that best fits the current operating point and from which the state-space matrices are computed. The state-space matrices are then used to compute the LQR gain for the current linearized model. The complete control diagram is shown in Fig. 4.

The third control method relies on an inequality index. The Gini index (see for instance Parkale & Nalbalwar, 2017) is specially interesting as it is used to assess sparsity in a sequence of values, which, in this application, can be identified with the cane spending time in the vertical position (a naturally goal configuration). Thus, high values of the Gini index may be interpreted as the cane spending more time at angles close to zero. Implicitly, this approach accounts for the fact that in normal operation controlling the cane for a constant zero angle is unrealistic. The user will always introduce disturbances that will tend to make the cane deviating from the zero angle. Other sparsity measures have been proposed in literature (see for instance De Villemeur & Leroux (2022) for a survey of inequality indexes, including for example multiple entropy-like indexes). However, the survey in Hurley & Rickard (2009) selects Gini’s index as the most adequate (the only that verifies six, allegedly intuitive, criteria described in the article). Similarly, Parkale & Nalbalwar (2017) also concludes by the superior consistency of the Gini index, hence providing additional support to the use in this project.

**Figure 5:**
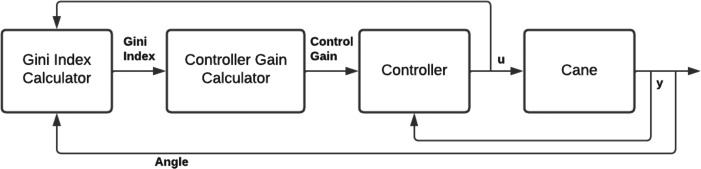
Integration of the Gini index in the low level control.

In the Gini-based controller, the control gain is adjusted according to the Gini index computed, in realtime, over a sliding temporal window. Every 11 s the Gini indexes for the angle and control variables are obtained and linearly combined. The combined Gini index, denoted *G* in the article, is compared with the previous value and checked for increase/decrease, and the control gain, *K*, then adjusted according to Eq. (4),

(4) \begin{eqnarray*}{K}_{k}={K}_{k-1}+\text{sign}({G}_{k}-{G}_{k-1})\,W\,{G}_{k}\end{eqnarray*}

where *k* is the time index and *W* is a constant vector of weight values that adjusts the effect of the Gini index on the controller gain. The control gain is adjusted depending on the Gini index variation, *i.e.,* if in an iteration the control gain is increased/decreased and the Gini index improved, the next iteration will vary the gain the same way, and if the index worsened the next iteration will vary the control gain the opposite way. Figure 5 shows a simplified schematic of the implementation of this method.

## Results and Discussion

This section presents three classes of results, obtained with two canes differing only in the control strategy. The first group of tests used an LQR-based controller. For the second group of tests, a gain scheduling stage was added, such that the LQR-based controllers are computed for linearized models that best fit the current state. The third group uses a version of both methods with real-time gain adjustment using the Gini index. This index belongs to the class of inequality indexes, often used as sparsity measures, (see for instance Parkale & Nalbalwar, 2017).

### LQR-based controller

Regarding the first group of results, previous tests with the two versions of the prototype (see Neves, Sequeira & Santos, 2021 and Neves, Sequeira & Santos, 2022), were conducted in cramped places but with smooth surfaces, and only the walking and standing portion of the movement was analysed. This article adds a new set of tests, where the device was tested on different surfaces, varying in smoothness and obstacles/debris, and in standing up/sitting down situations.

Most tests have a short duration due to the impairments of the users, which cause fatigue, pain and discomfort after longer periods of standing and walking, forcing frequent breaks that interrupt and limit the testing. Also, the majority of the tests were performed in the homes of the users.

**Figure 6:**
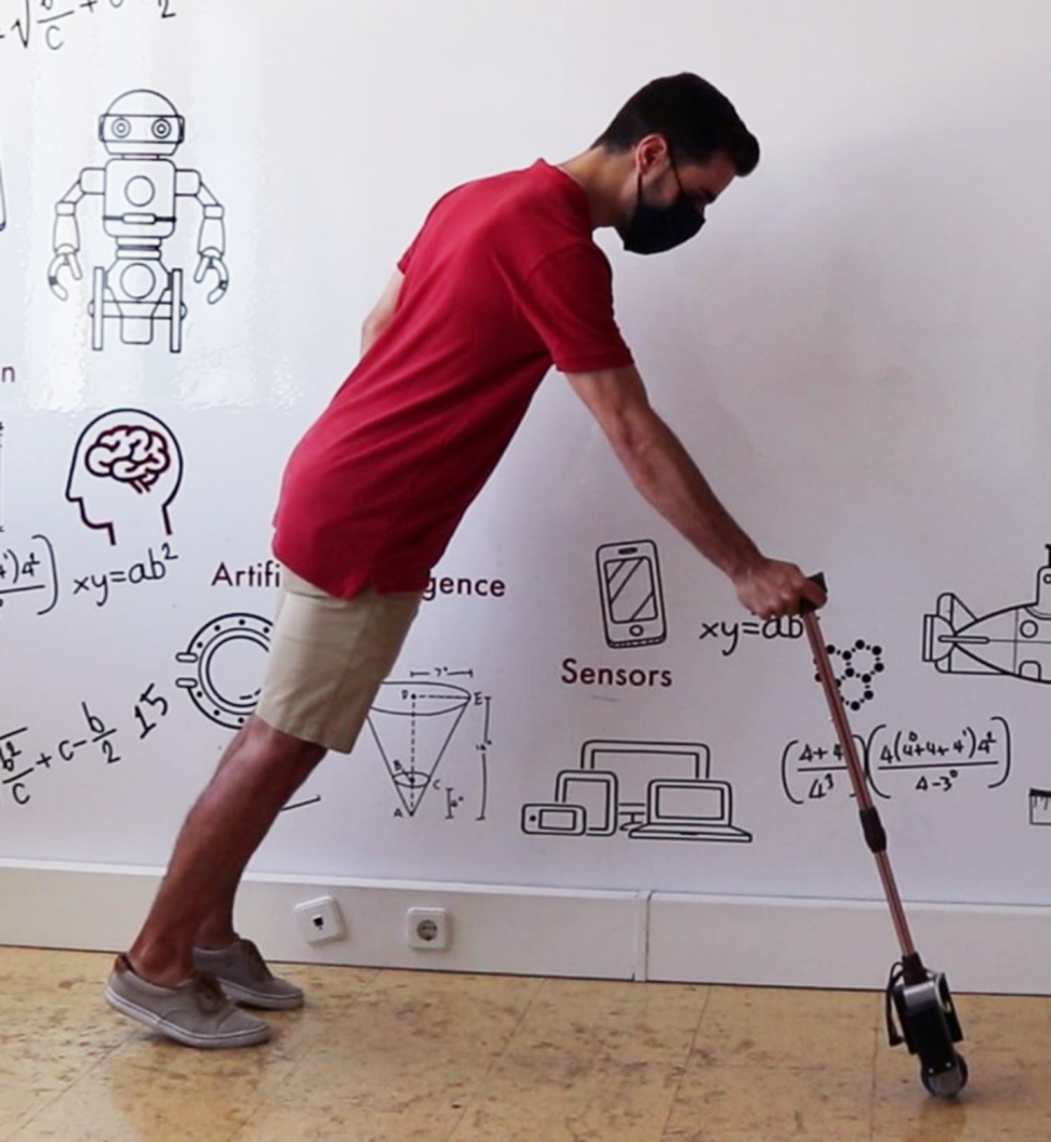
Analysis of the ability of the cane to bear the user’s weight.

Compared to the first version of the prototype, this second version has a more powerful motor, improving the control, and resulting in stable movement of the cane. This also improves the support given to the user, enabling the support of the full weight of an average user, as shown in Fig. 6.

In order to make an initial assessment of the cane’s capabilities, a test was carried out with an elderly user with motor skills greatly affected due to age. This user usually requires a walker for moving.

As mentioned in Sebastião et al. (2017) and Iosa et al. (2014), ageing and a sedentary lifestyle lead to problems in maintaining stability while standing and walking, thus increasing the risk of losing one’s balance and falling. The bipedal nature of humans results in an unstable system if not correctly controlled, and a third contact point is required to preserve stability, *e.g.*, in the presence of deficits of agility, balance, and cognitive fitness. In the elderly, this third point is often created by placing the cane instinctively in front of the body.

**Figure 7:**
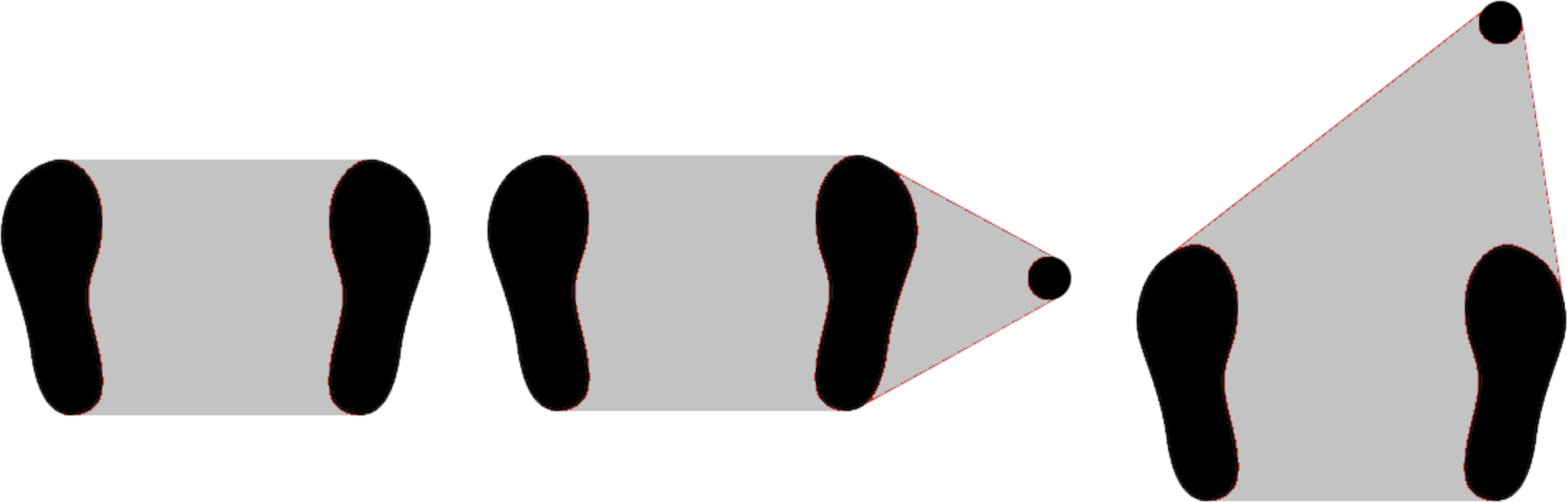
Support polygon area in different situations: no cane, cane at side, and cane at front.

The balance of a standing person can be described as the ability to maintain the projection of the centre of mass on the ground within the area delimited by the support points (support polygon) (Vadakkepat & Goswami, 2008). In cases where the balance control is affected, a solution is to increase the area of the support polygon with a cane or other support device. Placing this additional point in front of the user is a natural choice as it corresponds to the direction of movement and, therefore, the direction most prone to a loss of one’s balance. Figure 7 shows examples of support polygons in situations without a cane, with a cane at one’s side, and in front of the user. As mentioned before, the cane is controlled to an angle of −7^∘^, inclined towards the user.

**Figure 8:**
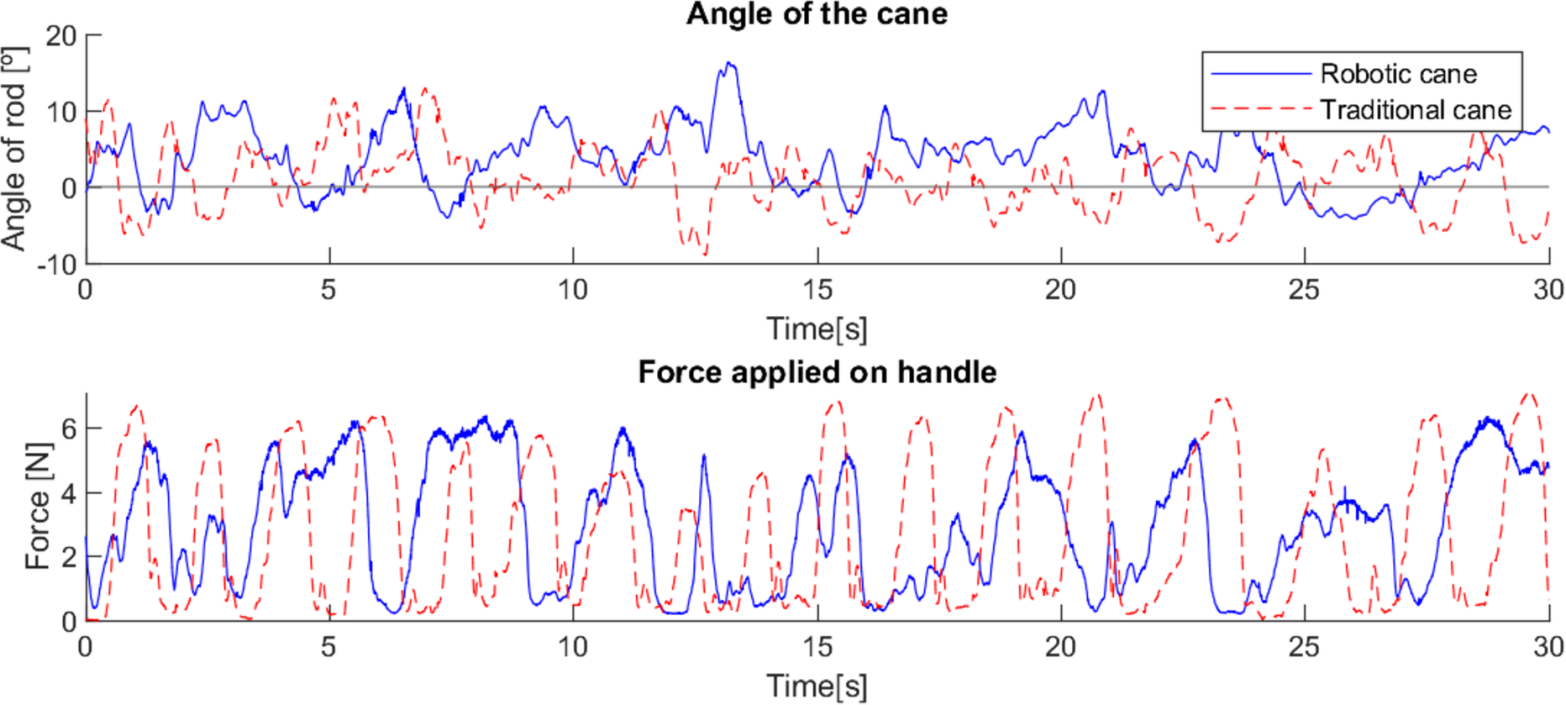
Differences in the force applied and the angle between robotic and traditional cane with an elderly user.

Comparing the robotic cane with the traditional one, in a trial with an elderly user, it is possible to observe that the former offers a more constant support to the user, with less major variations of force applied at each step and between steps. Figure 8 shows the test with the traditional cane where 17 peaks of applied force are observed; only 12 are observable with the robotic cane, and most major periods of force applied have a longer duration, proving that support is offered over a longer period of time, resulting in fewer moments of unsupported user movement. This difference is even more significant when comparing the standard deviations of the force applied, in Table 3, with the robotic cane presenting considerably lower values than the traditional cane, while the higher value for the angle is expected due to the constant control of the cane’s angle.

**Table 3:** Standard deviation with an elderly user.

	Traditional cane	Robotic cane
Angle (deg)	2.34	4.23
Force (N)	4.36	1.89

The next trials were performed with another elderly user, with left hemiparesis, flexed posture and slowed gait, and who used a traditional cane as a support device. The user was immediately comfortable with the prototype. After a short period for habituation, the trial started with a comparison between the robotic cane and his traditional cane in terms of the support given when getting up and sitting down in a chair. Despite being able to get up and sit in the chair with either cane, the user mentioned several times that it was easier to perform this movement with the robotic cane.

**Figure 9:**
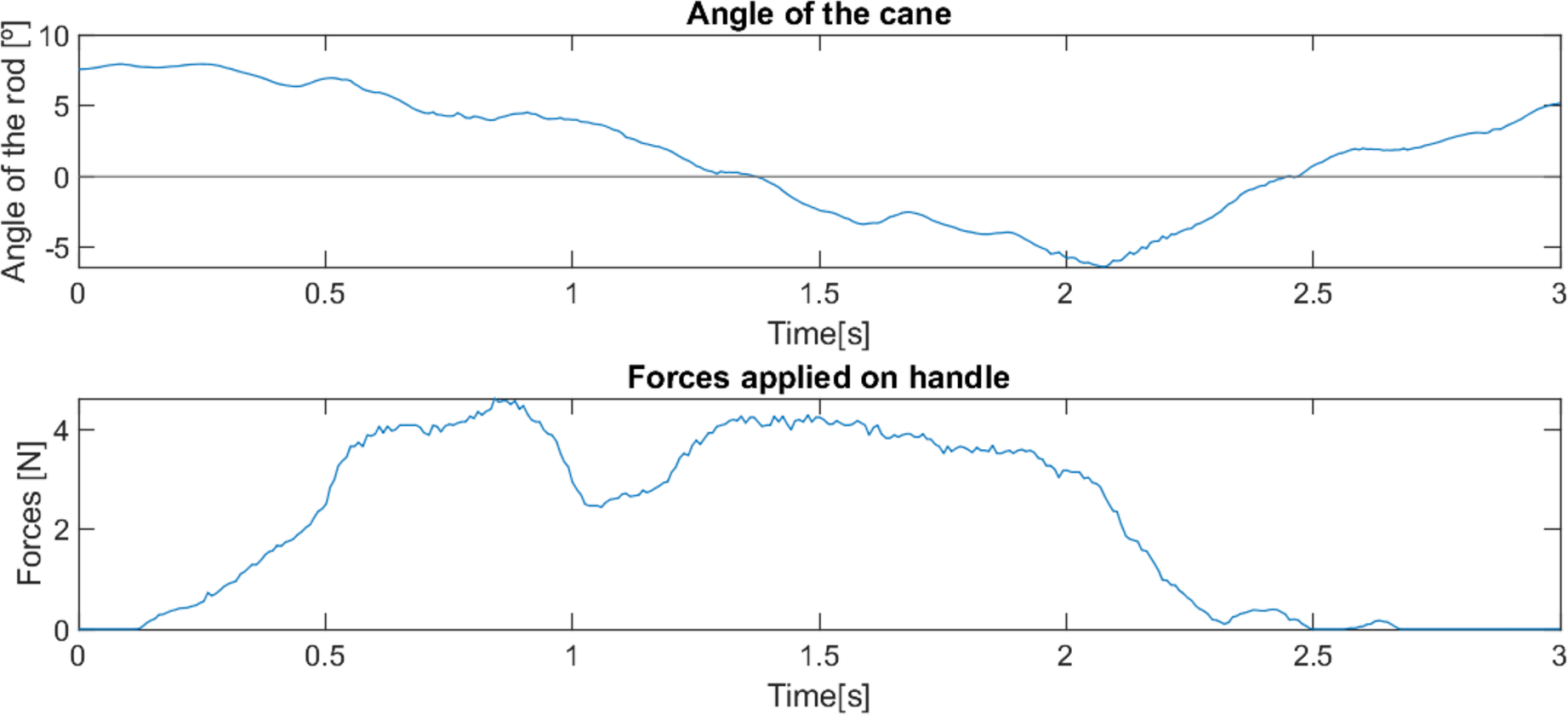
Behaviour of robotic cane with a reduced mobility user getting up from a chair.

**Figure 10:**
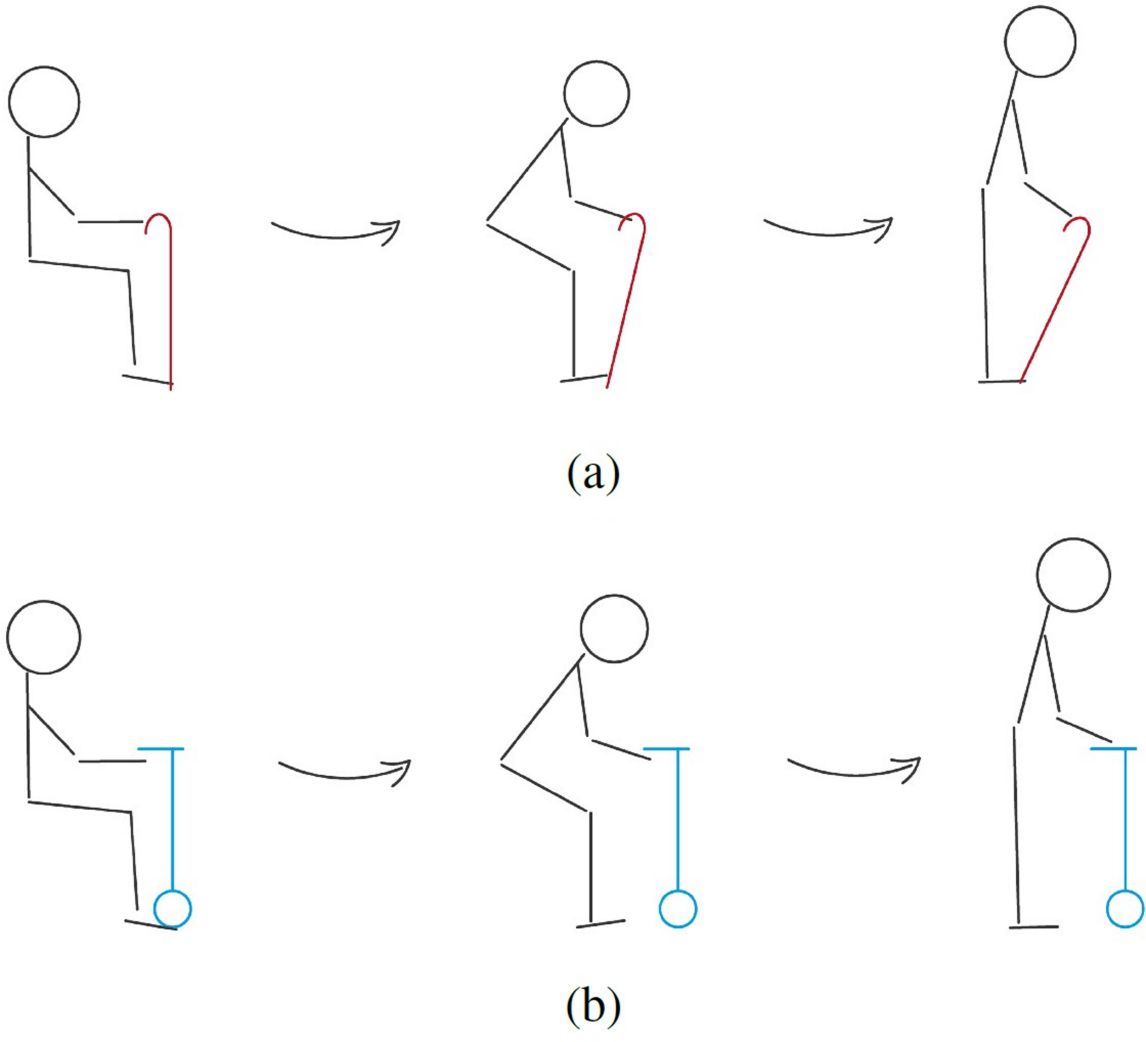
Representation of the behaviour of traditional (A) and robotic (B) canes with a user with reduced mobility getting up from a chair.

This cane is able to adjust to the variation of the user’s position, remaining stable and in the position where it offers more support (close to the vertical position), as can be observed in Fig. 9. The traditional cane, not adapting automatically, transitions to a position where it offers little support (inclined). Figure 10 illustrates the differences in the usage of both canes.

**Figure 11:**
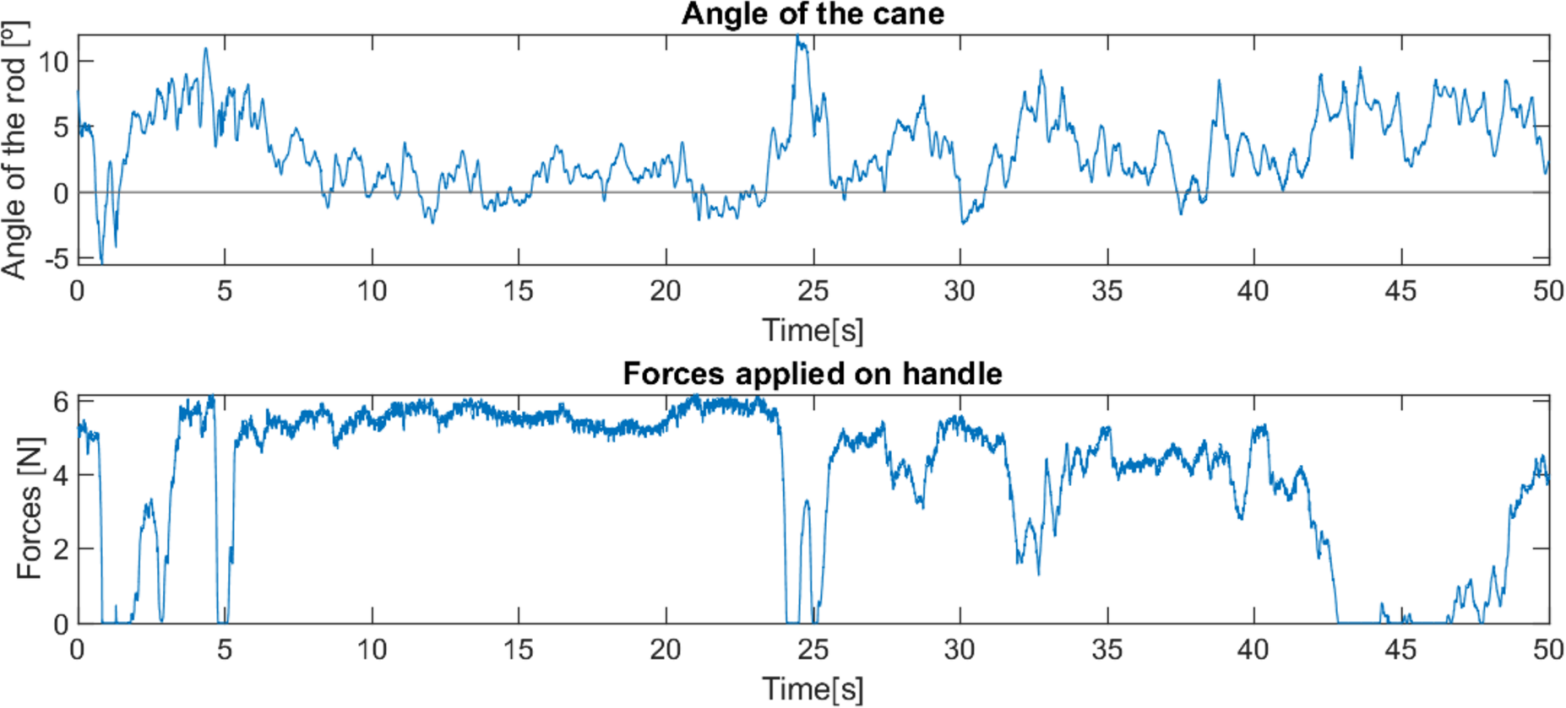
Behaviour of robotic cane with a user with reduced mobility on smooth pavement.

The first test was in a domestic indoors environment with a smooth floor. Due to the patient’s pathologies, his gait presents a very interrupted rhythm, where after each step there is a moment of pause, requiring great support throughout the whole movement (see Fig. 11). The cane was able to successfully keep up with the user’s pace, advancing and stopping as needed, and offering constant support. As the surface was smooth and without any obstacles, the displacement of the cane took place without any interference, and its behaviour was smooth and predictable.

**Figure 12:**
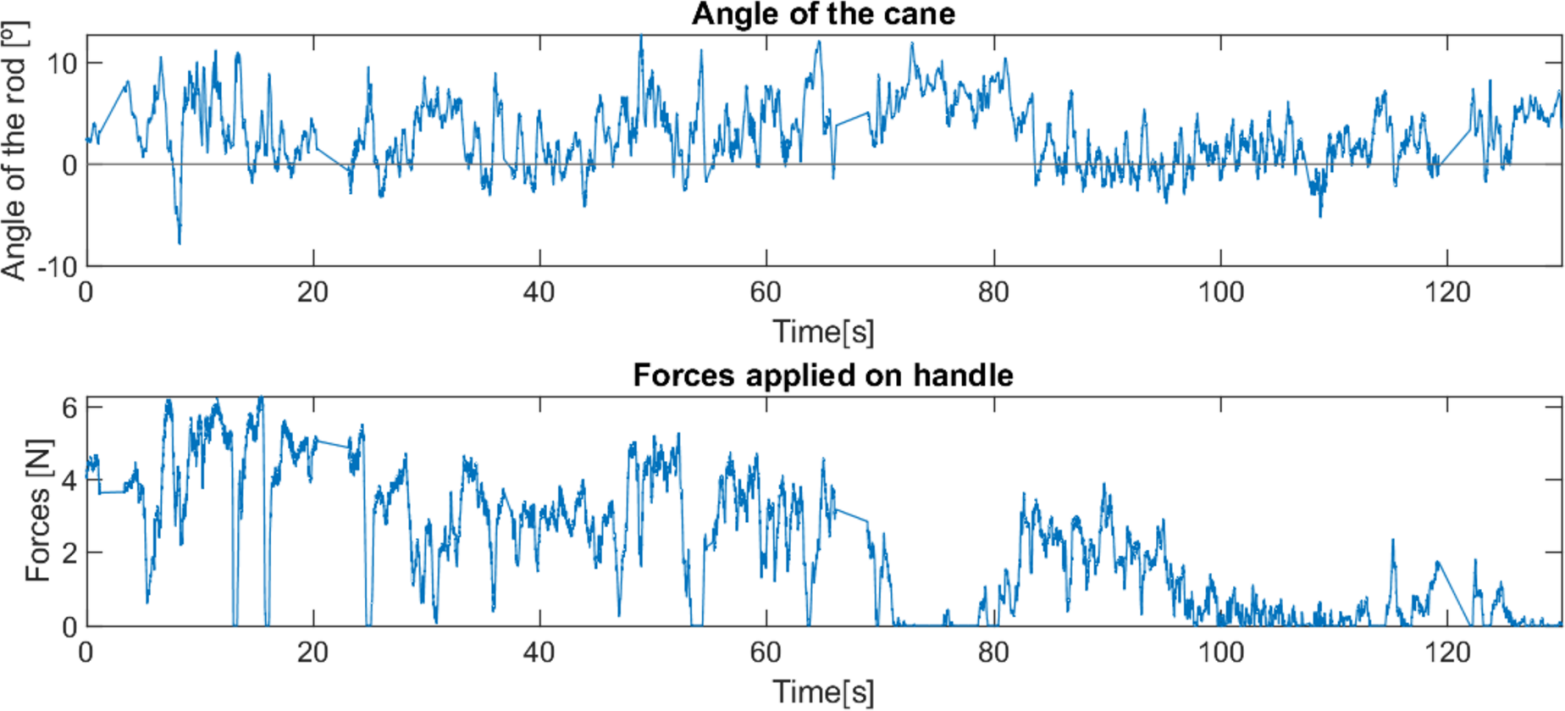
Behaviour of robotic cane with a user with reduced mobility on textured pavement.

The second test (see Fig. 12) was carried out on a floor with smooth reliefs (textured tile), outside the house. Since the textures of the surface were very subtle, they did not present any obstacle for the device, and, again, the device behaved smoothly and offered support to the user throughout the test.

**Figure 13:**
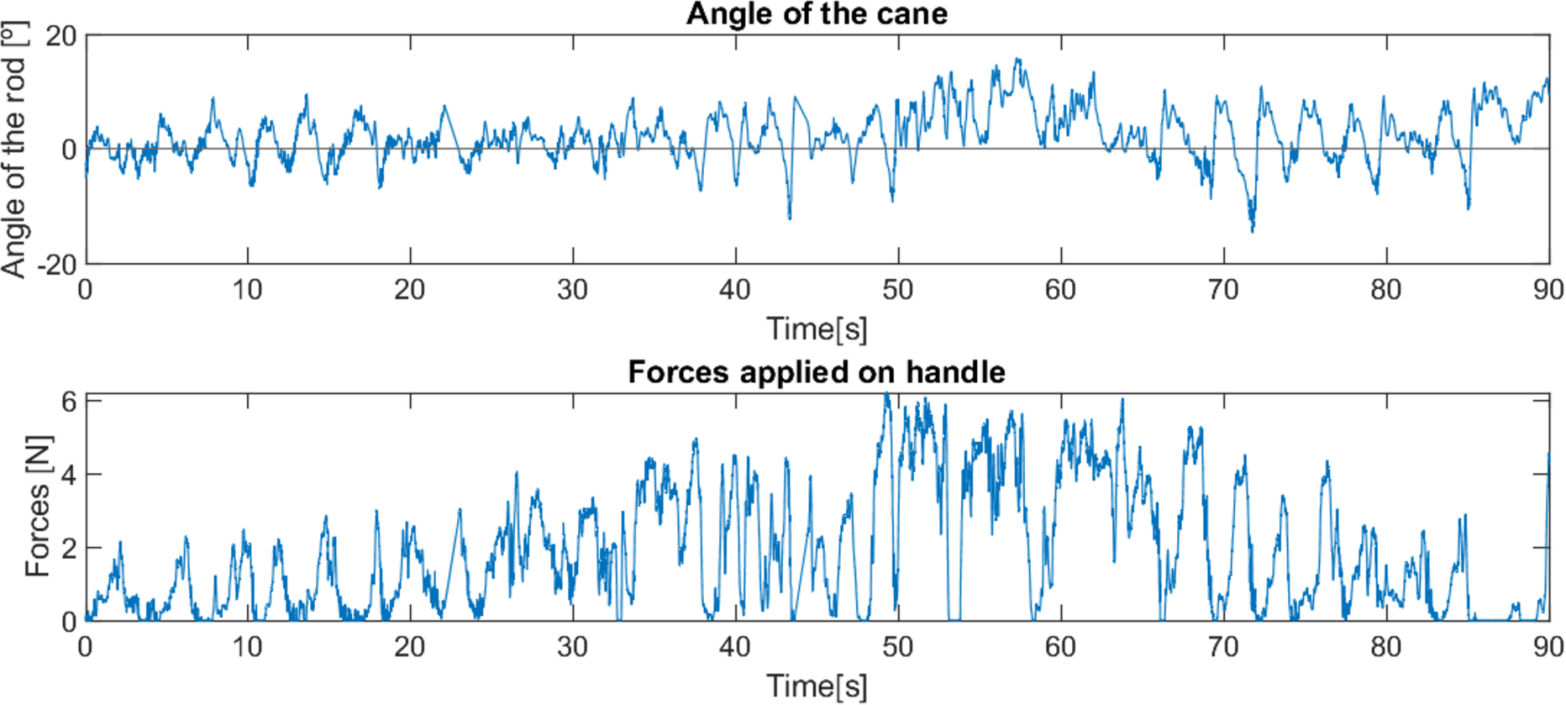
Behaviour of robotic cane with a reduced mobility user on a pavement with moderate irregularities.

Then the device was tested on a pavement with moderate irregularities (sidewalk in good condition, see Fig. 13). In this case, it was noted that the cane was momentarily delayed by some of the more pronounced irregularities, being able to recover proper movement in a reduced and adequate period of time. However, this led the user to raise the cane in some of these situations, in order to facilitate the continuation of the movement. This situation is mainly induced by the wheel used, which, despite being made of rubber, is solid and slick, leading to a poor adjustment to the irregularities of the pavement. The motor could generate the torque needed to overcome these obstacles, and, even with an inadequate wheel, the device behaved better than expected in this situation and, apart from the moments when the user lifted the cane, constant support was offered.

In the final part of this test, the pavement had a small slope, which also allowed analysing the behaviour of the cane in inclined areas. Again, the behaviour of the cane was smooth and, with the exception of occasional moments when the user lifted the cane, it offered support throughout the movement, although with greater variations due to the irregularities.

**Figure 14:**
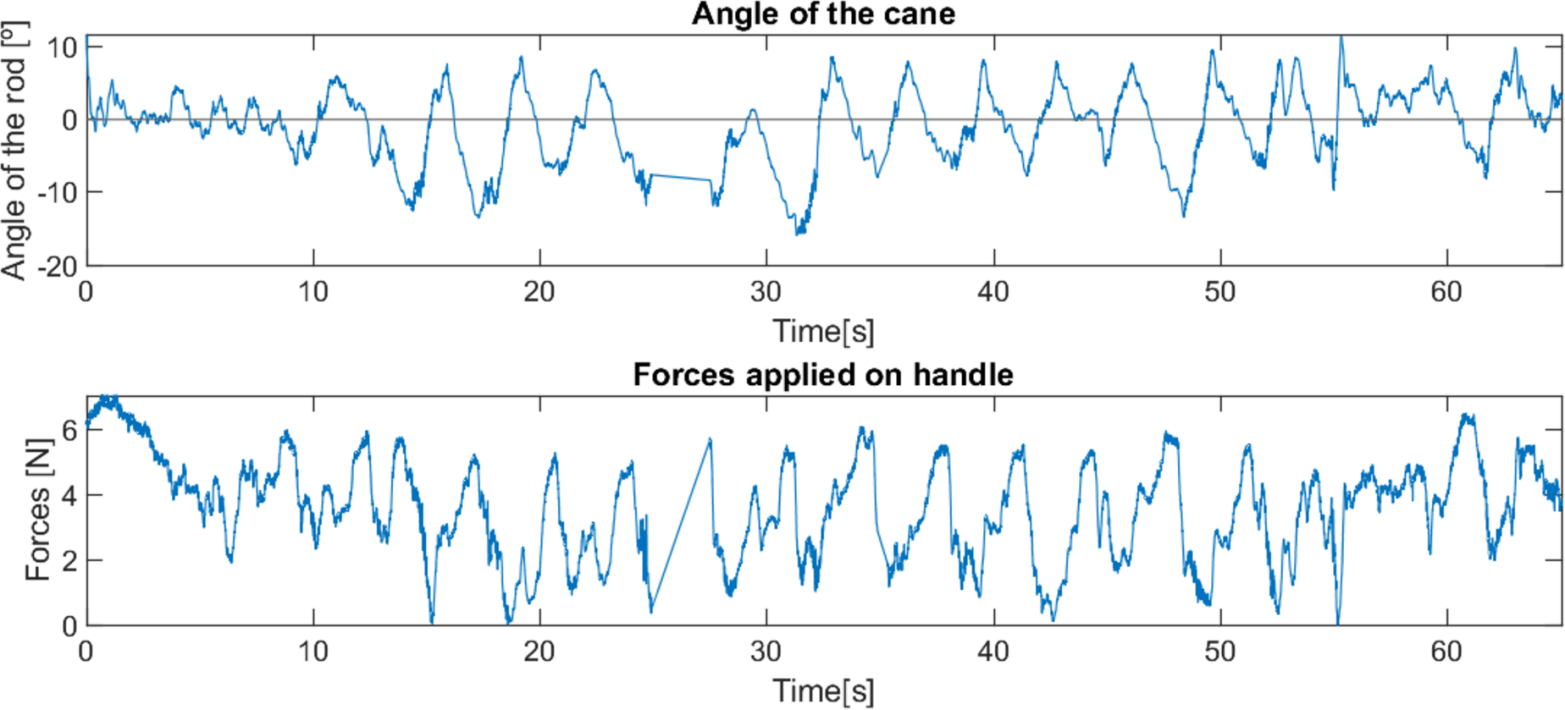
Behaviour of robotic cane with a user with reduced mobility on a pavement with debris.

The behaviour of the cane was tested on a regular pavement with small occasional obstacles (paved road, but with small stones and loose residues). The beginning of the test corresponded to an ascent, allowing, again, analysing of the cane in inclined areas (see Fig. 14). As with the previous test, there were some moments of difficulty in moving the cane, in which it was momentarily stuck due to the stones on the pavement. However, it always ended up being able to free itself from these obstacles without the need to be lifted. The device’s behaviour was very similar to the previous test, presenting a smooth movement and offering support throughout the movement, with variations due to the obstacles.

The user’s opinion was quite positive, mentioning on several occasions that he preferred this cane to a traditional one and that it facilitated his locomotion.

Subsequently, 32 tests were carried out with this control method with other users in different situations and stages of reduced mobility, which made it possible to confirm that the device controlled by this method is very intuitive, allows a quick habituation, and offers enough support so that most users felt safe and supported, often showing interest in the device and preferring this cane to a traditional one. It also allowed confirming an hypothesis that has been constant in all the work carried out, which is the enormous diversity that exists in the characteristics of the need for support of each user, mainly in situations where there is a greater disability in locomotion and, therefore, a need for greater support.

**Figure 15:**
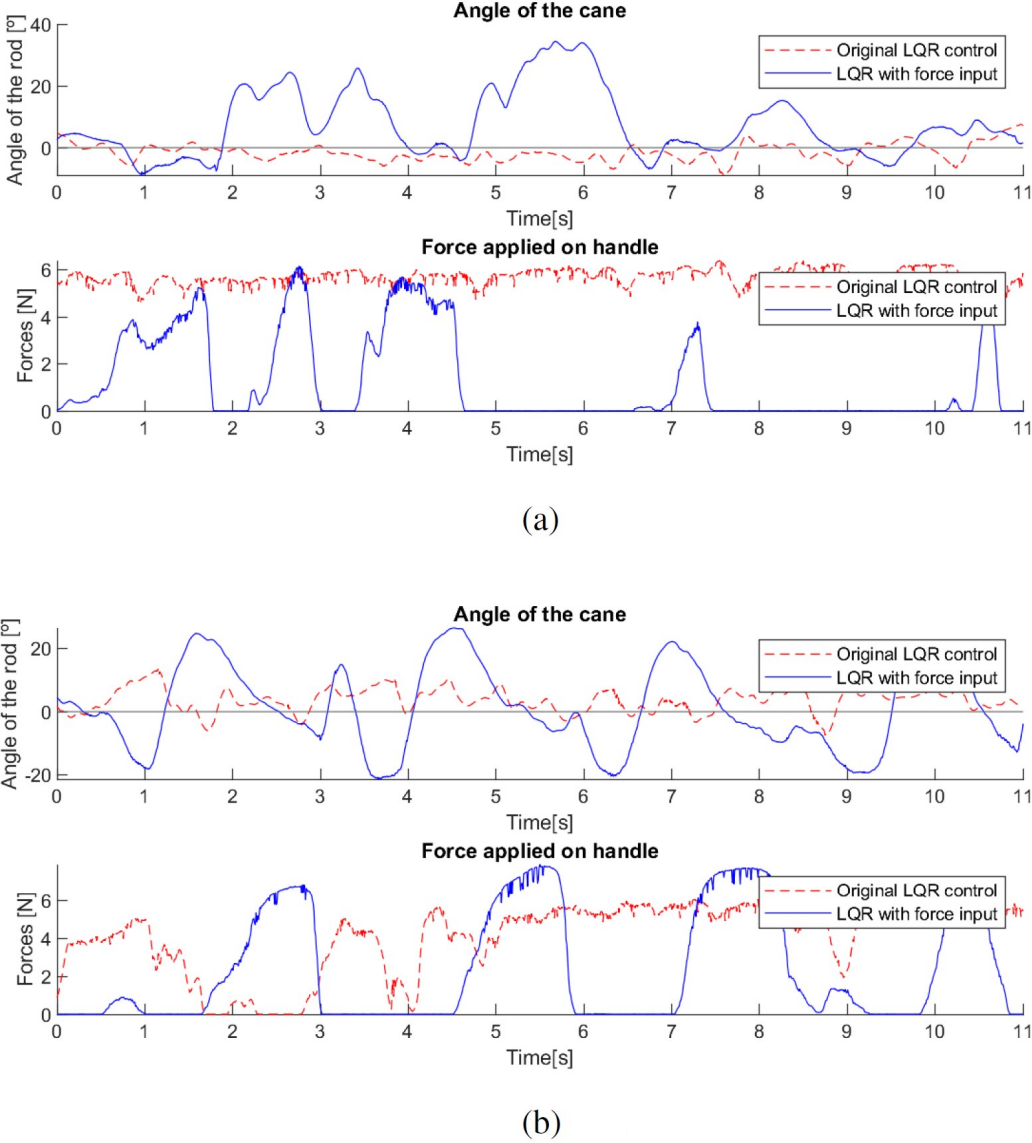
Behaviour of the robotic cane with first (A) and second (B) control methods.

An alternative control method was tested, with the force applied by the user being used to adapt its position and angle, in that it moves like a traditional cane, with a swinging motion with each step of the user. As with the previous method, this method was tested with impaired users (see Fig. 15).

Due to time constraints and the fatigue of the users, the tests carried out were of short duration, not allowing full adaptation, especially with the cane controlled by the latter method, which meant that only short periods of the tests corresponded to correct use of the canes. Hence, the shortness of the period of time presented in the graphs shown in Fig. 15.

Both users suffer from similar pathologies, but have different levels of impairment, resulting in different needs for support, with the second user requiring more support than the first, leading to the occasional use of a traditional cane, while the first user does not use a cane.

These results show a big difference in the appropriate target group of users for each control method. The latter method offers greater support in cases where it is required (due to more severe mobility impairments, as in the second test) and is also more intuitive and functional with users who already have the knowledge and training in how to correctly use a traditional cane, since it mimics this behaviour. This also implies that the support won’t be as constant, even if more intense, since the user will apply the majority of the force each time the injured leg takes a step. In situations where the user requires less intense but more constant support and is not accustomed to using a traditional cane, as in the first test, the cane appears to perform better when controlled by the first method, with a more constant angle and force, even if not much higher than the peak values of the force involved in the second method.

### Controller with gain scheduling

This section illustrates results with two identical, one controlled by the LQR method with force input explained in the previous sub-section, and the other controlled by this gain-scheduling method, with a total of 7 users with restrained mobility (user 1, Lumbar osteoarthritis; user 2, Diabetic neuropathy; user 3, Diabetes, multiple falls (possibly hip arthrosis); user 4, Ischemic stroke (caused left hemiparesis); user 5, Stroke (caused left hemiparesis); user 6, Bilateral knee prosthesis; user 7 diabetic neuropathy, decreased visual acuity).

The needs of each user for support were significantly different since their medical conditions were different (different genders, different pathologies and different ages—most test subjects were elderly, and thus prone to age-related illnesses (Sebastião et al., 2017; Iosa et al., 2014)). As before, all the tests were conducted under strict supervision of healthcare professionals. The participants were duly informed and consented to participating in the trials.

The participants in the tests belong to a group of users with mobility problems being followed by a physical therapy doctor. The aim is to rehabilitate motor skills, learn habits of correct gait, and train how to act in fall situations. One of the habits they learn in this therapy is how to move their arms while walking, as users often keep their arms fixed during locomotion, which is an incorrect habit, since the arms should swing at the sides of the body in a movement opposite to the legs (specially with users that use canes).

One of the differences observed between the two control methods was that as the first method explained requires an oscillating movement of the arms, it was much more natural for users who had already had training on how to move their arms during locomotion *versus* users who had not had yet this training. For users that had not yet had experience or training with correct walking gait and cane use, the first method has shown a slow learning curve, requiring a long habituation, while the second method allowed a faster learning curve, with a natural first use by these users.

**Figure 16:**
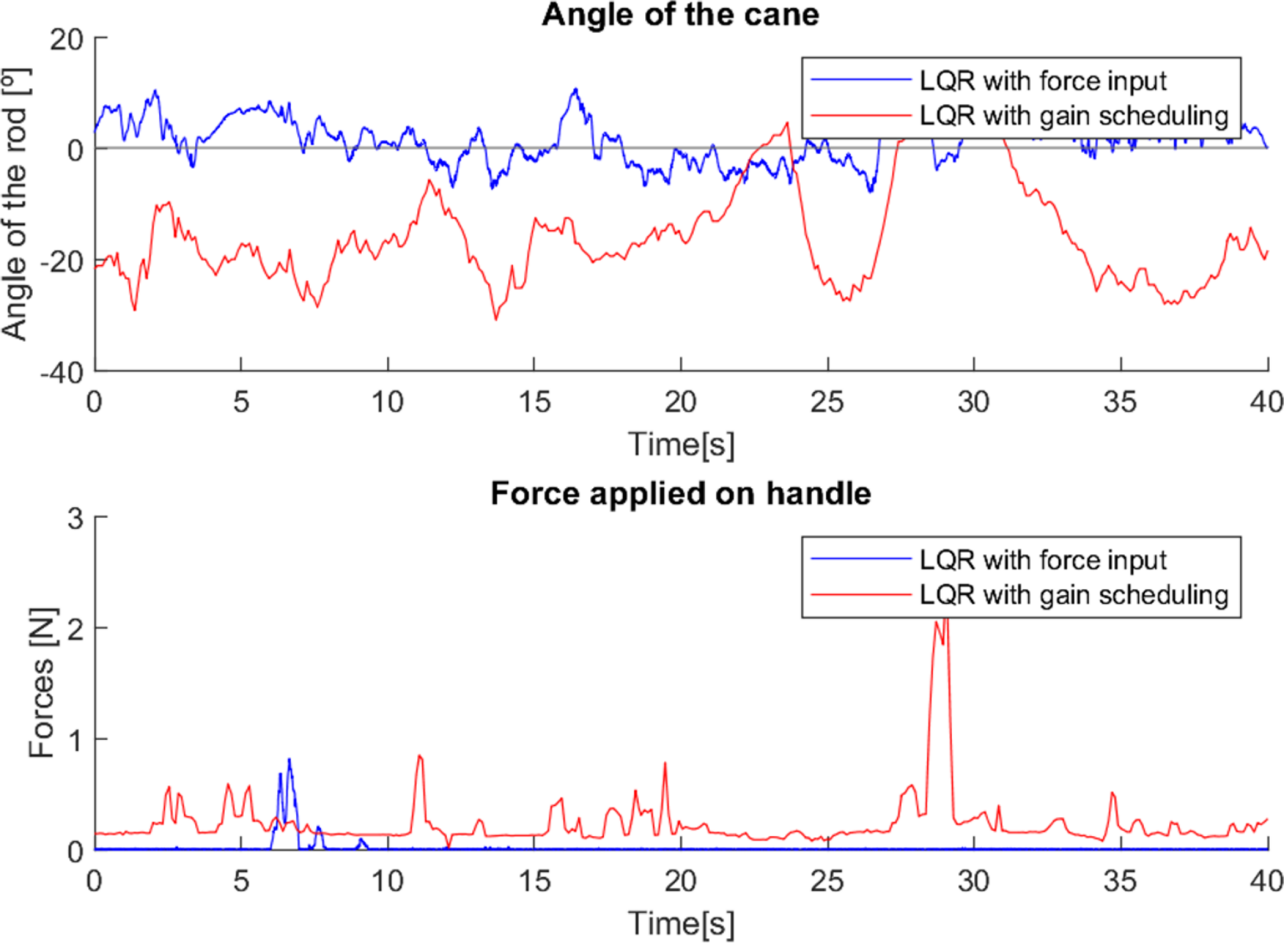
Test with user 1.

In the first test (Fig. 16), the user had not yet had any training in how to correctly walk and use a traditional cane, and did not feel the need for a stronger support, so the force applied to the canes was minimal, and the angle varied little in both cases (walking with both canes relatively vertical). Since the first control method adjusts the movement of the cane with the force applied, in cases like this where the force applied is small, the behaviour of the cane was similar to what was studied in Neves, Sequeira & Santos (2022), in which the cane was only controlled to maintain a fixed angle close to the vertical, observable in this test. The second method, with gain scheduling depending on the system state, allows for a better adaptation of the cane’s behaviour to the situation, hence the larger variations of the angle around −20° (which is observable in most trials).

**Figure 17:**
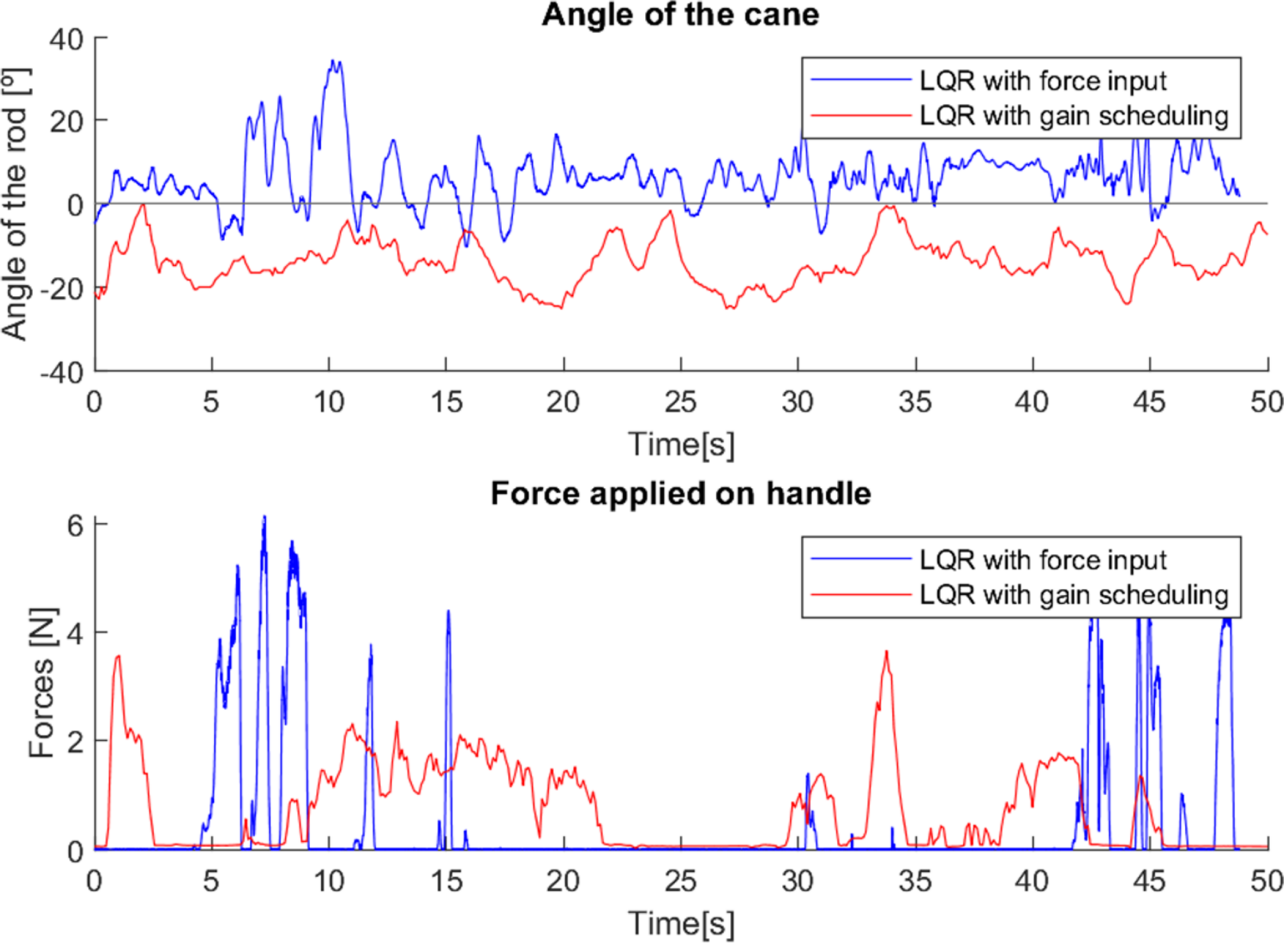
Test with user 2.

In the second test (Fig. 17), it is possible to observe two intervals (5–15 s and 42–50 s approximately) in which the cane with the first control method (LQR with force input) was correctly used (with back and forth swinging and strong support in each step, similar to the use of a traditional cane). However, in the rest of the test the cane was used vertically (the user was already trained in how to correctly use a traditional cane, but in most of the test she used it as she felt more comfortable).

The cane with the second control method (LQR with gain-scheduling) was used with relatively little force exerted, and with a near-vertical movement.

**Figure 18:**
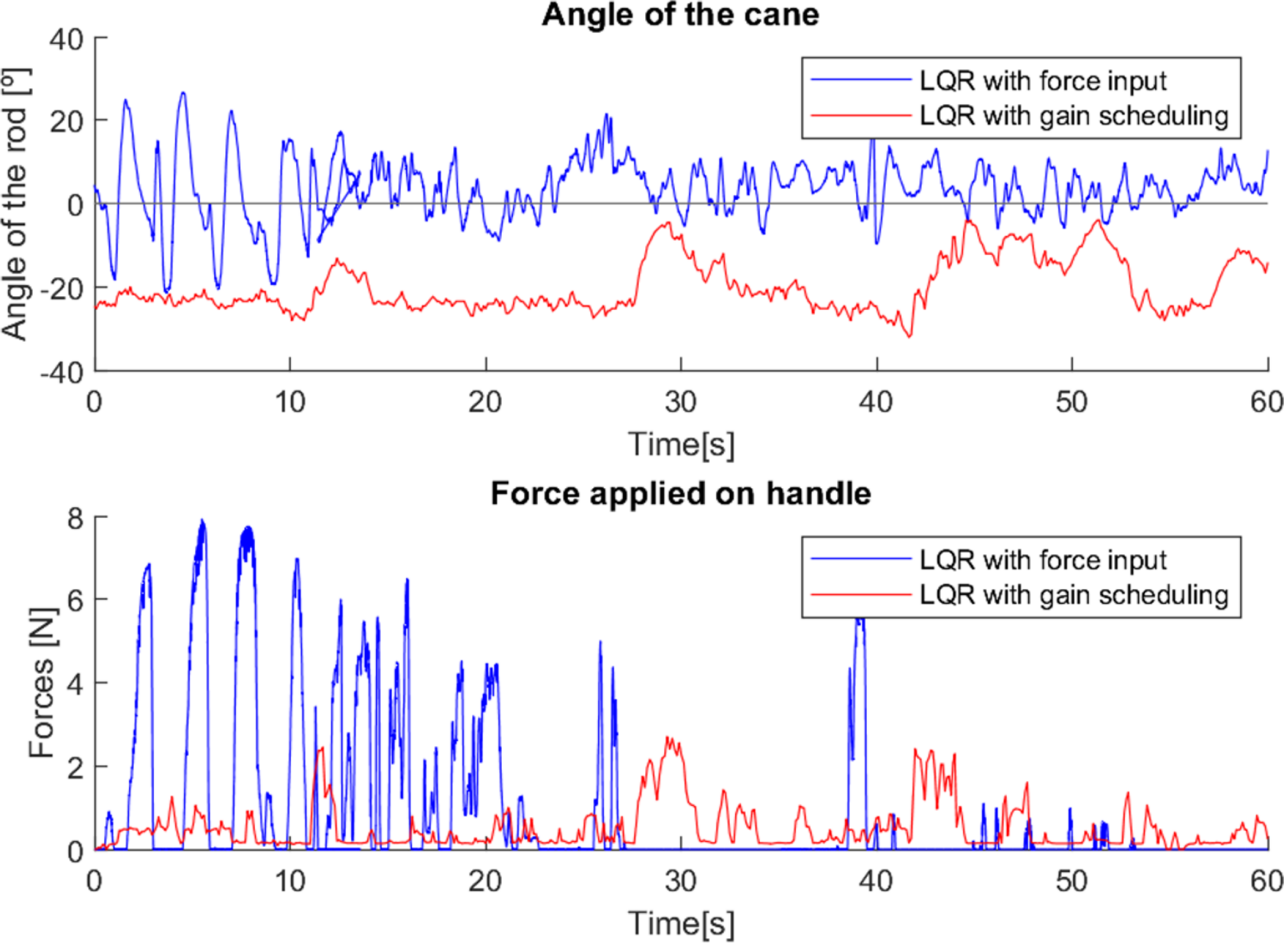
Test with user 3.

As in the previous test, in the third test (Fig. 18), it is possible to observe two moments (0–15 s and 38–40 s approximately) in which the cane with the first control method was correctly used. However, in the rest of the test the cane was used vertically (again, the user was already trained in how to correctly use a traditional cane, but in most of the test he used it as he felt more comfortable).

The gain scheduled cane was used with relatively little force exerted, and with a constant movement of approximately −20°, except when the user turned around, and therefore applied a “stretch” on the cane, creating a short variation of the angle (11–13 s, 27–30 s, 57–60 s), and in the interval 42–53 s he stopped to speak, moving his arms as he spoke, which caused inconsistent variations in the angle.

**Figure 19:**
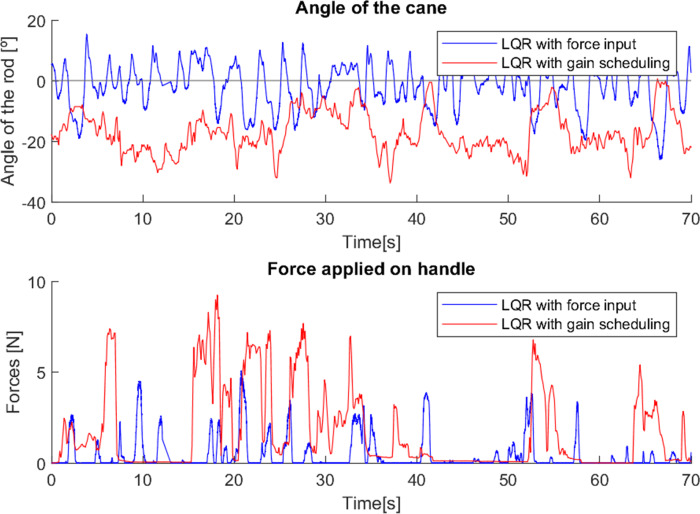
Test with user 4.

The user in the fourth test (Fig. 19), had few mobility difficulties, and was able to use both devices quite correctly.

In the cane with force input, the variation of the angle and the force applied to the cane with each step is easily observed, as it is used in a similar way to a traditional cane (as it should be).

However, it was the cane with gain scheduling that offered the most support. The variation of the angle was less intense, and the support was not as constant, but when it was necessary to provide a lot of support, the cane was able to provide it. It should be noted that, sometimes, more intense forces made this cane interpret the moment as a fall, which caused it to block the movement and induced a sudden variation in the angle, since the user was not expecting the cane to stop (52–56 s).

**Figure 20:**
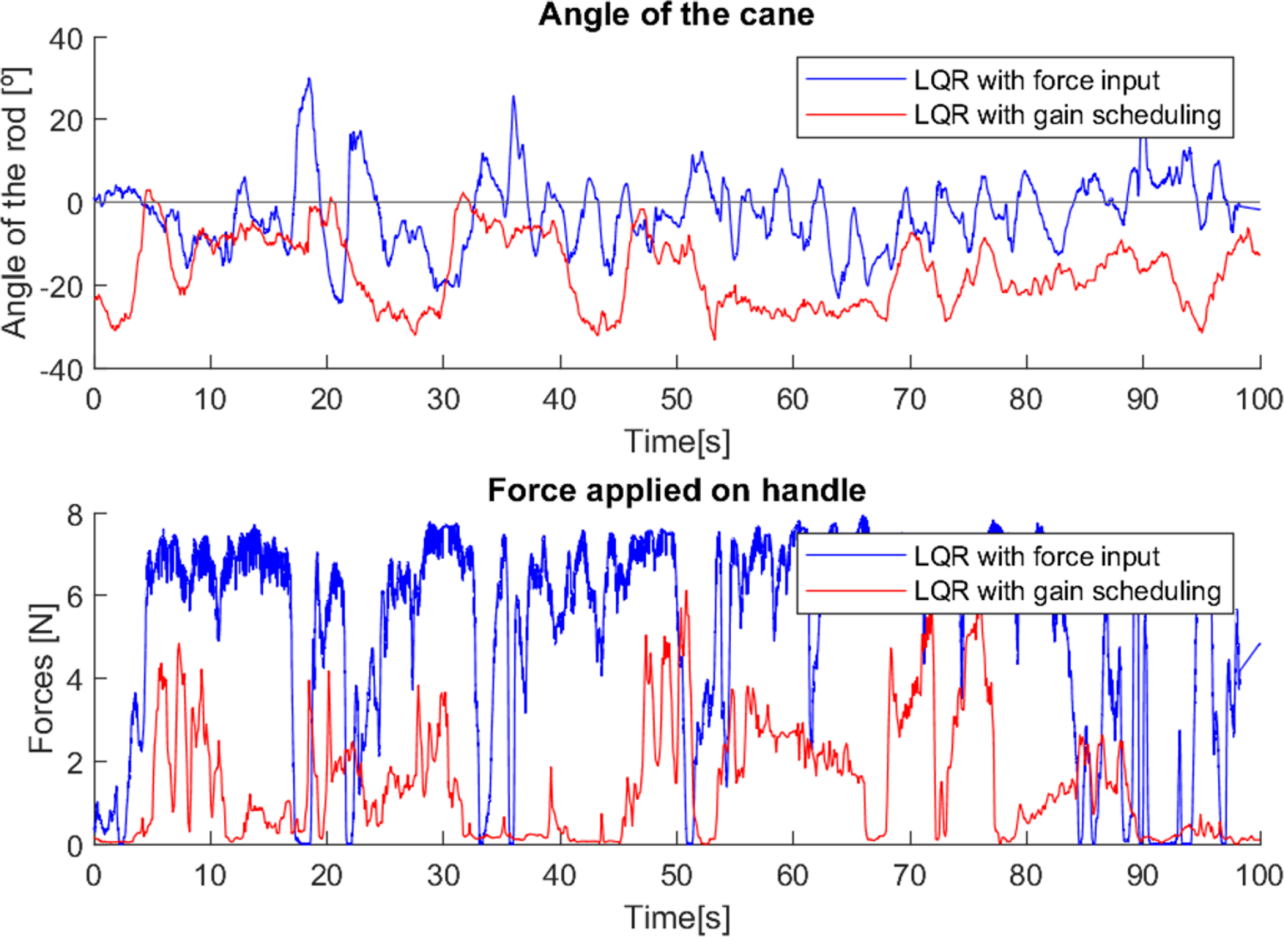
Test with user 5.

The fifth user (Fig. 20), had greater motor difficulties, so a more intense support was necessary. As such, the cane with force input allowed more intense support for the user, observable over the whole of the test. Since the cane was used correctly, as a classic/regular cane, it suffered larger variations of angle than the cane with the gain scheduling.

**Figure 21:**
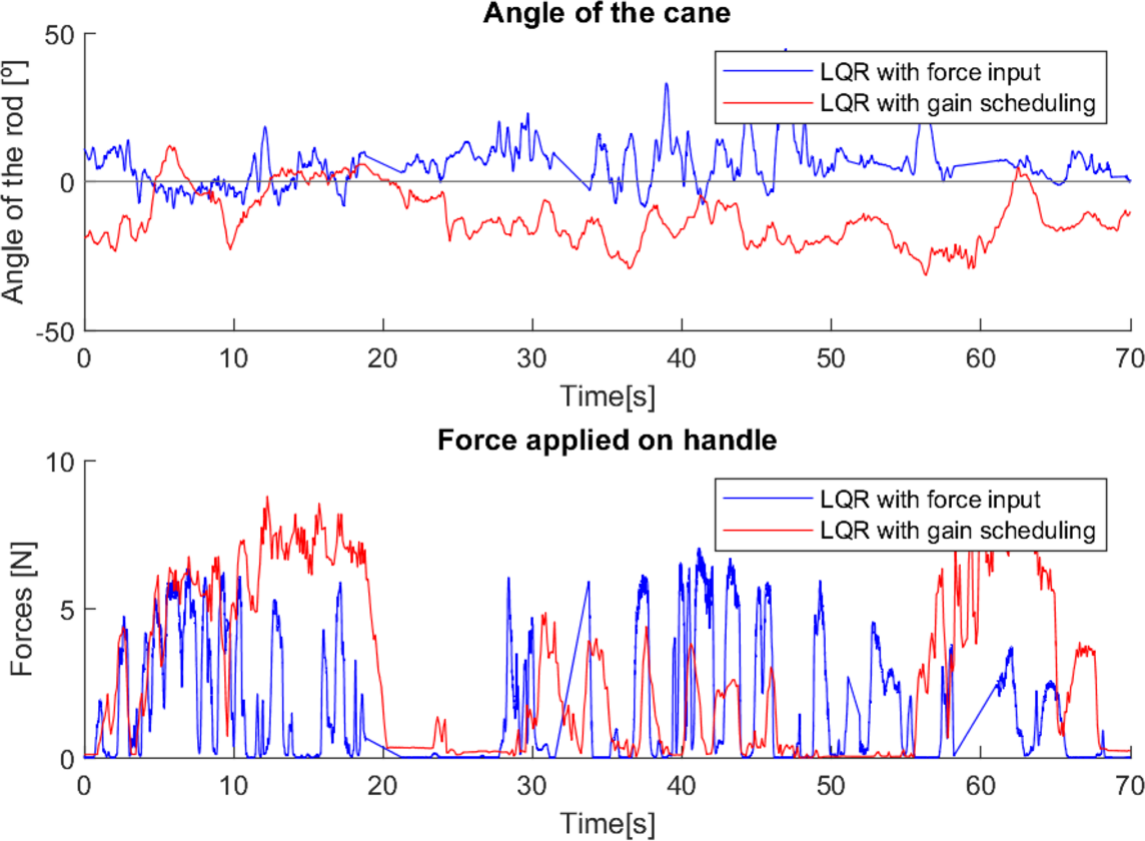
Test with user 6.

The sixth trial (Fig. 21), presents challenges in the analysis of the data obtained. This user forcefully and unwittingly grabbed the handle of the cane due to numbness in the hand, which caused several moments of false detection of falls by the second control method due to the high force read by the sensor on the handle. Despite this, two moments can be observed (3–20 s and 56–65 s) in which the force applied to the cane with the second control method has a more intense and constant value when compared to the first method.

**Figure 22:**
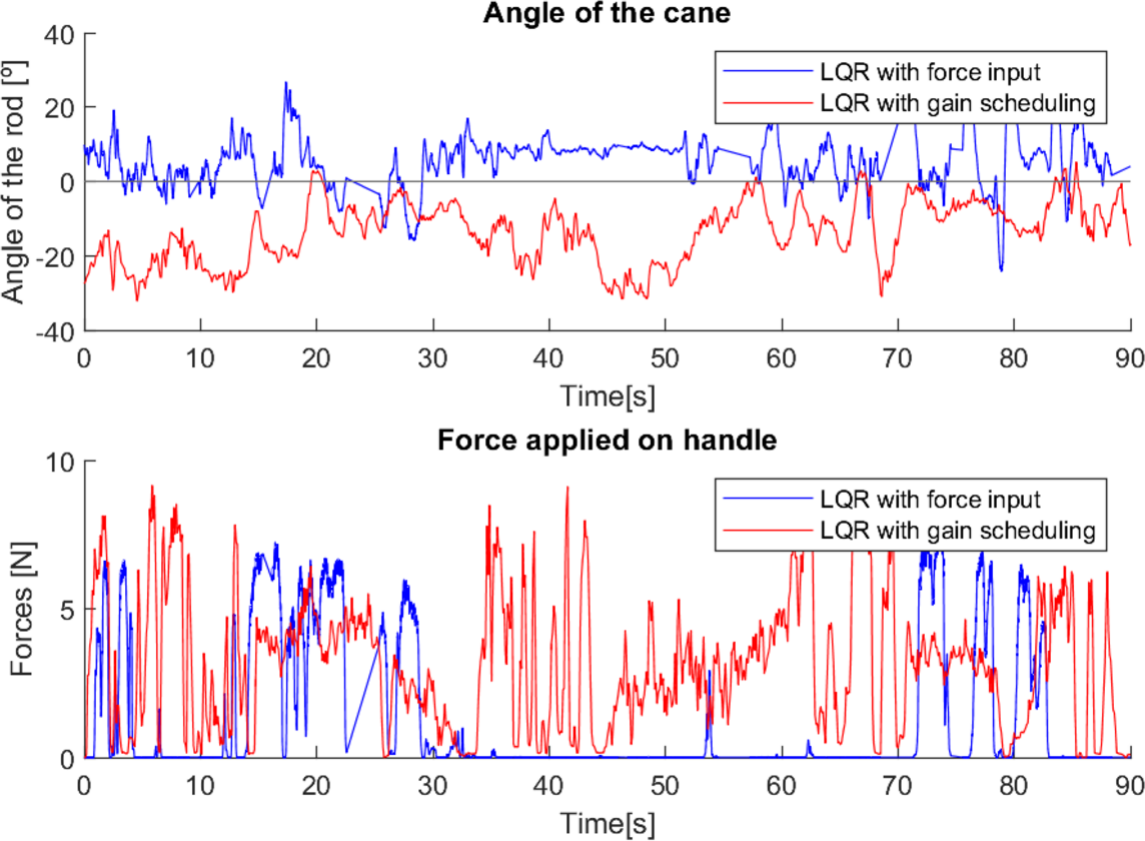
Test with user 7.

As in the first test, the seventh user (Fig. 22), had not yet had any training in how to correctly walk and use a traditional cane, resulting in a wide variation of the force applied and angle of the canes. Besides this, the second control method showed longer intervals of more intense force applied, indicating increased constant support.

The above tests show that the first control method tends to offer greater support to users who are already used to traditional canes and who have a greater need for support. However, it requires a longer period of habituation and a more thoughtful use, while the second control method offers more constant support, mainly to users who have never used mobility aids and who need less intense support; it only needs a shorter habituation period and provides a more natural use of the device. The tests also show that the requirements of the users of the locomotion for support vary. This is aligned with results reported in the literature, *e.g.*, Vadakkepat & Goswami (2008), that stability requirements depend on the user and environmental conditions.

Finally, two tests were performed with users without motor difficulties, to serve as a reference and be compared with the results obtained previously. Both users of these two tests were young, without any condition that compromises their mobility, and needed a long habituation time with the canes in order to use them correctly.

**Figure 23:**
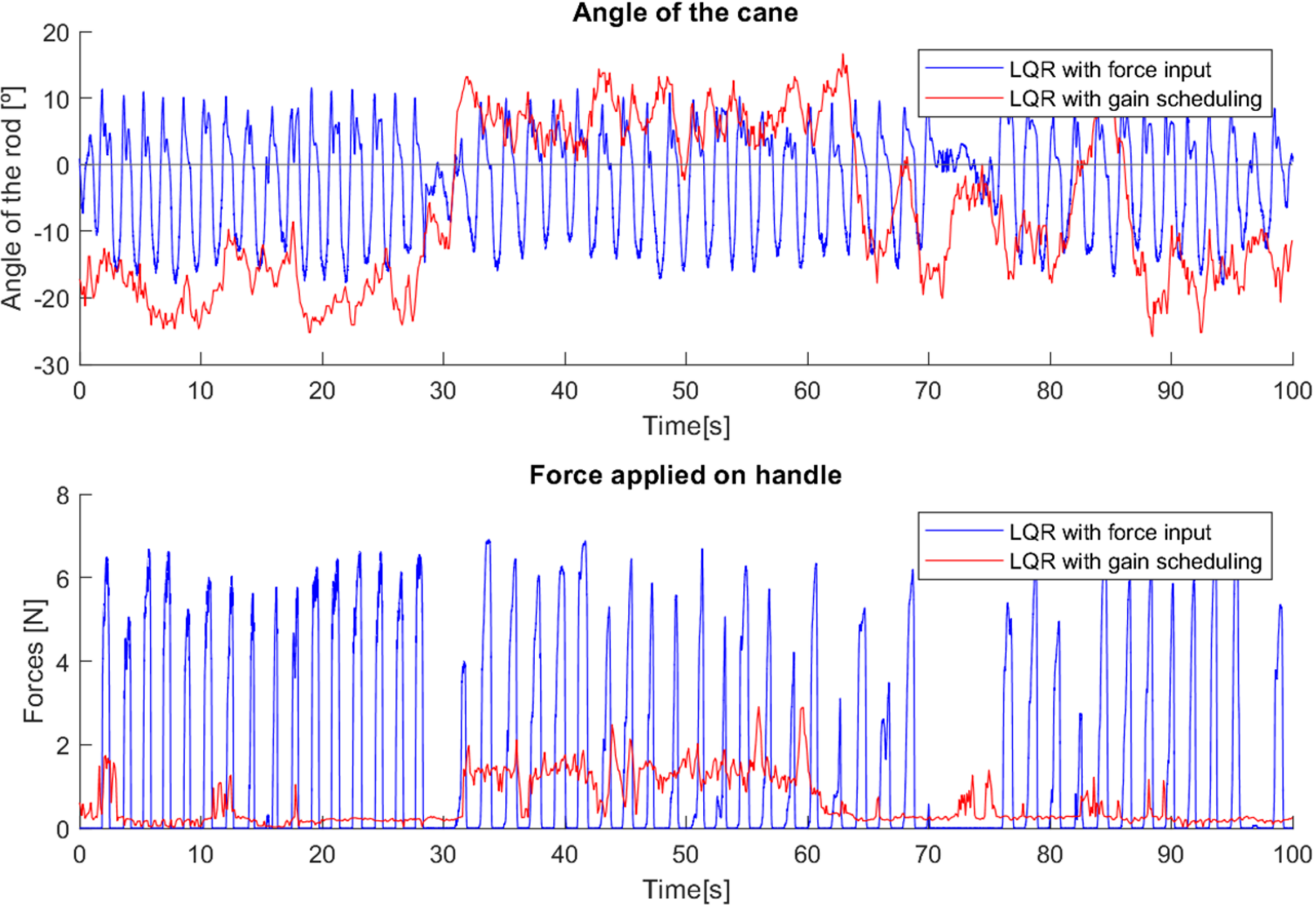
First test with a user without mobility problems.

**Figure 24:**
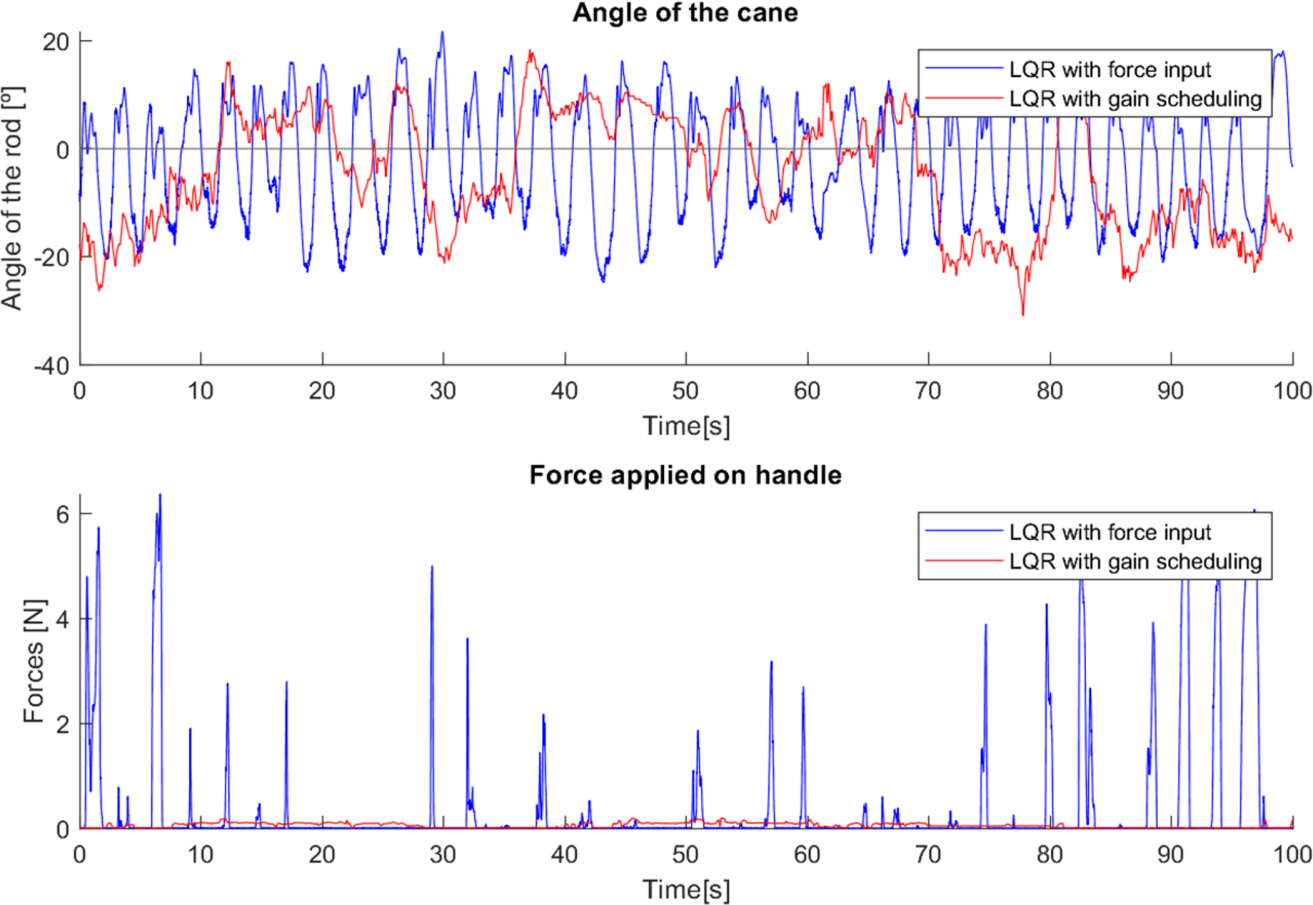
Second test with a user without mobility problems.

As can be seen from the tests (Figs. 23 and 24), the difference between the two control methods is even more noticeable. The oscillating movement introduced by the first method is clear, with a rocking movement in each step and a peak of force applied, corresponding to the moment of support. On the other hand, the second method provides a much more constant movement, with only a few variations resulting from the control adapting to the user’s movement. The applied force, despite not peaking as high as in the first method, shows less variations throughout the movement.

As these tests represent the correct use of canes, they present characteristics very similar to previous cases where users with reduced mobility used the canes correctly (*e.g.*, tests 2, 3 and 4). This suggests that with a longer habituation period to teach users how to use the canes and allow them to become familiar with the process, both canes would most likely be able to have a greater and more adequate effect.

The differences between the two control strategies become evident when inequality indexes are used to assess the data sequences obtained. The Gini index is specially interesting as it is used to assess sparsity in a sequence of values, which, in this application, can be identified with the cane’s spending time in the vertical position (a natural goal configuration). Thus, high values of the Gini index may be interpreted as the cane’s spending more time at angles close to zero. Implicitly, this approach accounts for the fact that in normal operation controlling the cane for a constant zero angle is unrealistic. The user will always introduce disturbances that will tend to make the cane deviate from the zero angle. Other sparsity measures have been proposed in the literature (see for instance Irawan & Santoso, 2019), including entropy-like indexes, or Hurley & Rickard (2009), including Hoyer and *pq*-means indexes). However, the survey in Hurley & Rickard (2009) selects the Gini index as the most adequate (the only one that verifies the 6, allegedly intuitive, criteria described in the article). Similarly, Parkale & Nalbalwar (2017) also concludes that the Gini index has superior consistency, hence providing additional support to its use in this project.

Table 4 shows the values of the Gini index for the sequences corresponding to the orientation of the cane and the control applied to the motor wheel (both these sequences can have negative values and hence the Gini index is applied to the absolute values in order to preserve the usual interpretation, De Battisti, Porro & Vernizzi (2019); Ostasiewicz & Vernizzi (2018)). Though the lengths of the sequences differ, since they correspond to similar experiments the corresponding Gini indexes are comparable.

**Table 4:** Values of the Gini index.

	Orientation		Control	
	Cane 1	Cane 2	Cane 1	Cane 2
Test 1	0.50904	0.29836	0.48201	0.24211
Test 2	0.38884	0.27097	0.38799	0.44213
Test 3	0.44663	0.18598	0.41844	0.13918
Test 4	0.41002	0.2349	0.48174	0.49297
Test 5	0.41046	0.36213	0.36169	0.26449
Test 6	0.36914	0.32826	0.38826	0.40967
Test 7	0.37402	0.32433	0.43674	0.5396
Test 1 (normal user)	0.39373	0.28707	0.49549	0.12486
Test 2 (normal user)	0.32376	0.35676	0.45030	0.20661

The values of the Gini index for the orientation angle of cane 1 show an increased value for test 1 but otherwise the values are comparable among all the tests. Similarly, for cane 2 the values of the index are comparable among the tests. The difference between the values for each cane shows that the control strategy for cane 1 leads to the cane’s using a wider range of angles and increased sparsity.

As for the control signals of cane 1, the values of the Gini index are similar among the tests. However, for cane 2 these show a significant variability among the tests, which, we hypothesize, can be identified with the pathologies of the users, namely those involving strong physical disturbances since this cane adapts its control to the user’s input. Extensive tests are scheduled for the near future to assess this hypothesis.

The Gini index for the orientation angle clearly shows that the usage of the two canes is consistently different, *i.e.,* the values for cane 2 are lower than those for cane 1 in all tests. The index for the control signal shows in tests 2, 4, 6, 7 higher values for cane 2 than cane 1, indicating that the controller for cane 2 performed differently.

### Controller with Gini index based tuning

**Figure 25:**
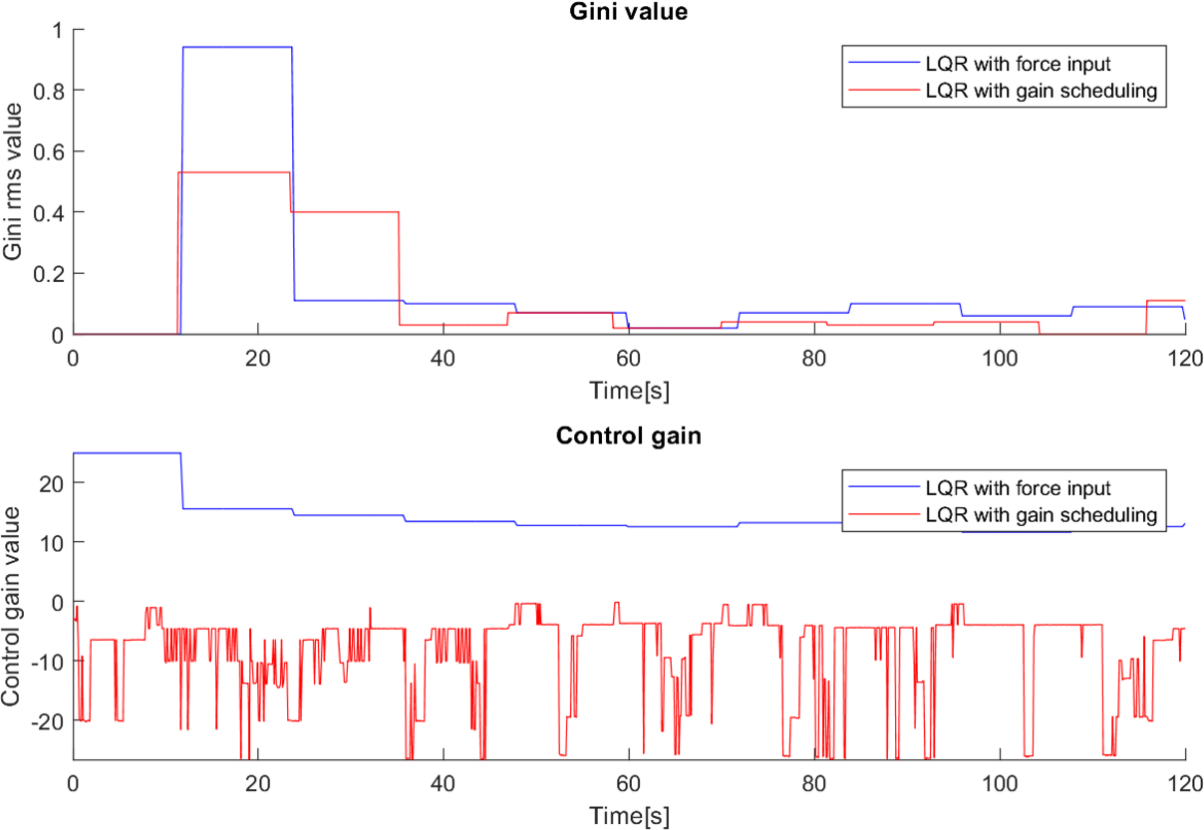
Effect of the Gini index on the controller gains.

The observation above suggest that the properties of the Gini index may be useful to tune the controllers. This allows the adjustment of the control gain in real time, targeting the improvement of the Gini index and smoothing the behaviour of the cane. This is noticeable in Fig. 25, where both canes with the Gini-based implementation were tested and in both cases the system adapted their controller gains to minimize the Gini index.

During the testing with the first cane, it was clear that the Gini index method adjusted the controller gain to the user’s movement, *i.e.,* when the user’s movement became slower the Gini index leads to a gain reduction, and vice versa. This suggests that the Gini index can also be a metric for the comfort of the user. In a situation where the system gain is inadequate to the user’s movement, it leads to unnecessary movements of the cane. For example, a gain higher than necessary will make the cane move faster than the user and therefore have to go back a little after each step, creating unnecessary variations in angle and control and reducing the comfort of the user. If the gain is adjusted to the user’s movement, these variations are avoided, improving the Gini index.

**Figure 26:**
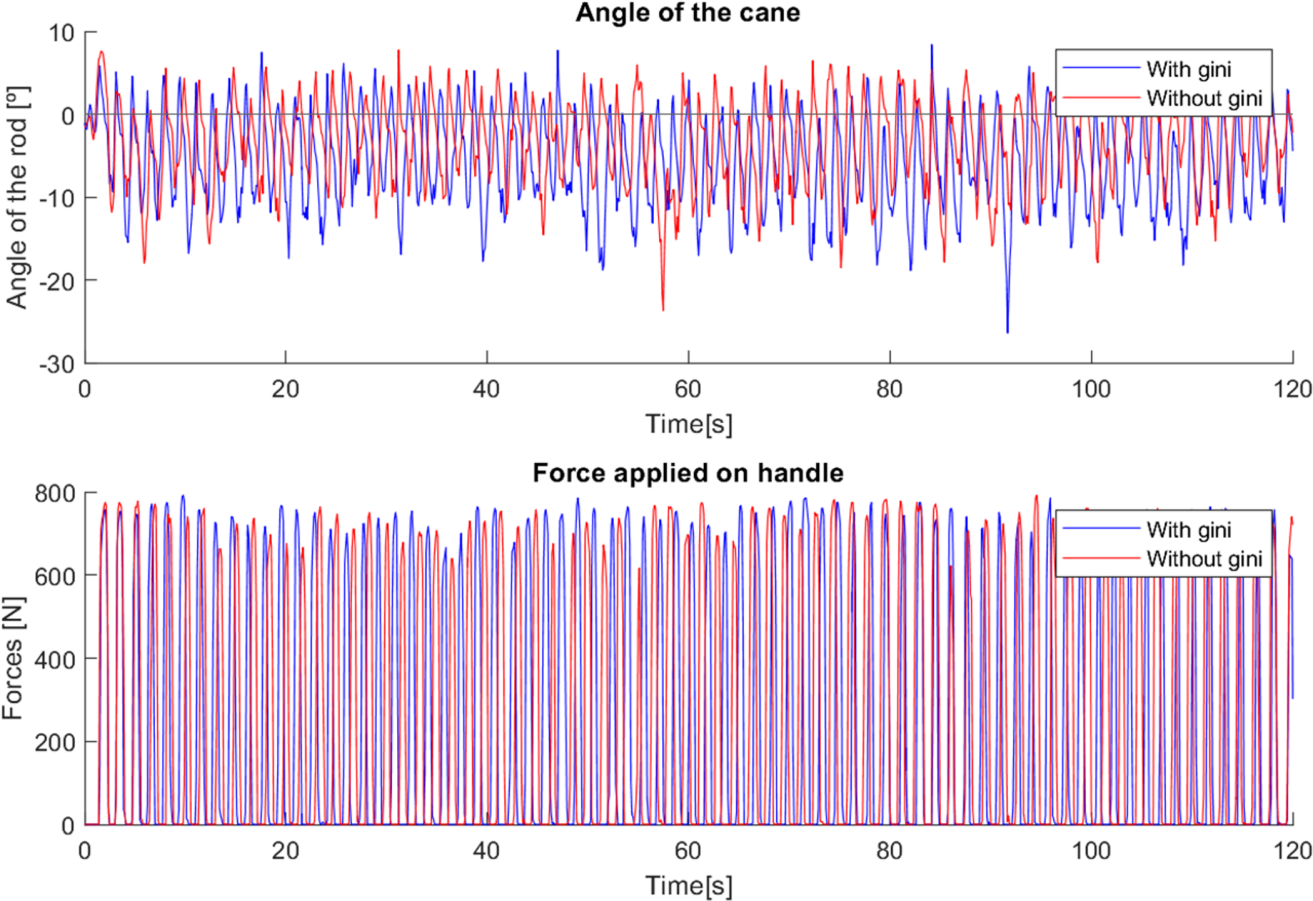
Test comparing first cane with and without Gini index.

This effect is visible in Fig. 26 in the variations of the angle, which start out similar in both cases but over time, since the system gain is too high for the user’s movement, the gain is reduced, leading to a slower response of the cane to the user’s gait, allowing it to remain longer at a negative angle, showing more negative angle variations and following the movement with improved comfort. The mean angle in the case without the use of the Gini index was −3.37^∘^, while with its use it was −5.68^∘^, which is a notable difference considering the implementation.

In the case where the gain was higher, the cane had a faster response, which did not allow such a comfortable slow movement. Using the Gini-based control, the values of the force are slightly higher in most steps than without the use of the Gini index. The tests with the Gini method also presented a few moments of intense jitter, derived from the practical limitations of the prototype, such as backlash in the motor gears and in the wheel shaft fittings, which were corrected with a low-pass type filter applied to the control signal.

**Figure 27:**
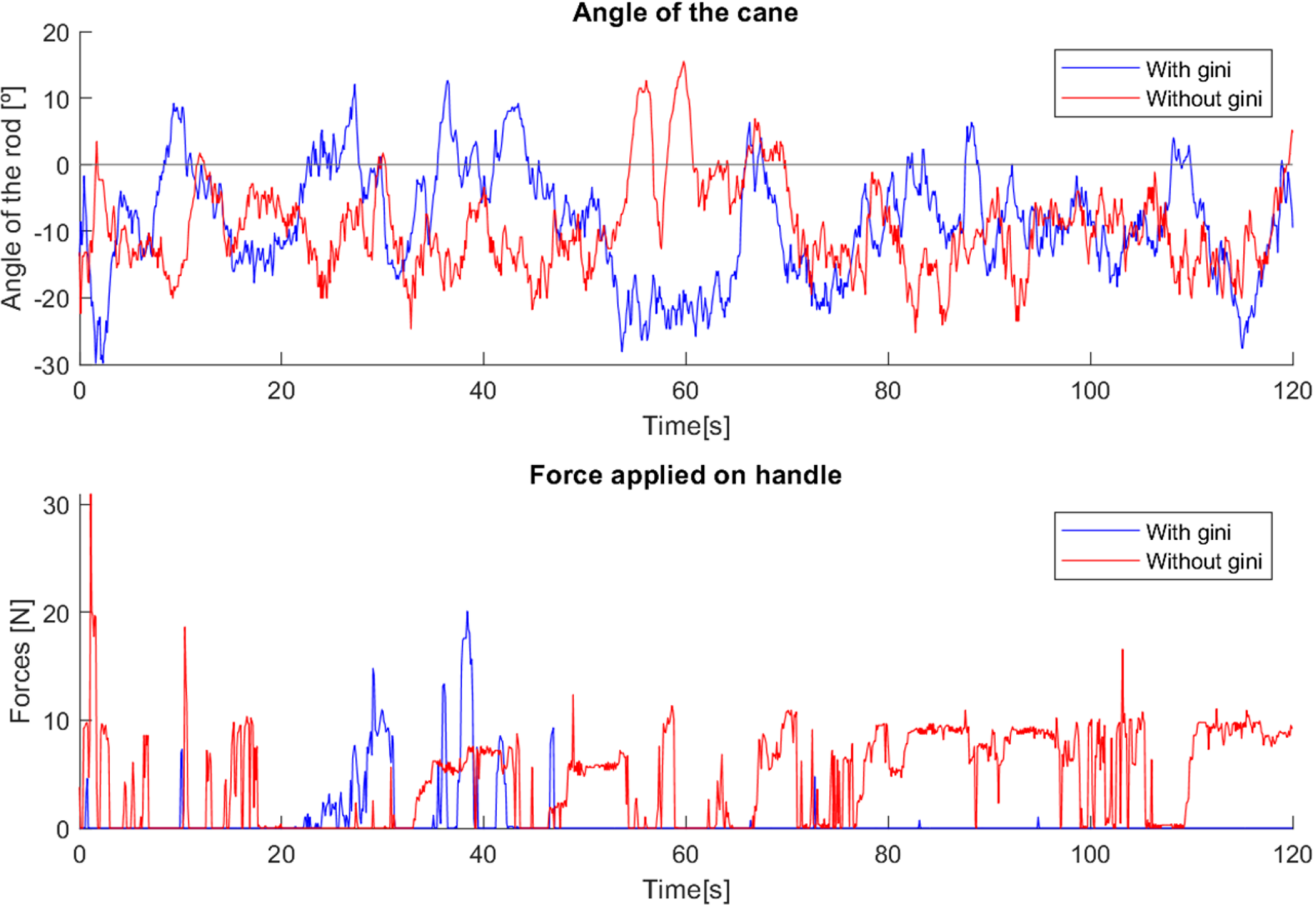
Test comparing second cane with and without Gini index.

As with the first cane, in tests with the second cane the most noticeable difference was in the angle, as seen in Fig. 27. The version with the Gini implementation shows an angle closer to zero (the mean angle was reduced from 4.444^∘^ to 1.205^∘^), meaning that the Gini method was able to keep the cane closer to the stable vertical position. The standard deviation of the angle was slightly reduced (from 4.164^∘^ to 3.603^∘^).

The force applied in the test without the Gini method presents periods of longer duration when compared with the version using it. We hypothesize that these variations result from differences in the grip by the user, though additional testing is required for confirmation.

## Conclusions

The results obtained so far, though not yet allowing statistically valid conclusions, strongly suggest the viability of the project (data from users covering a large spectrum of pathologies is required to ascertain the ones getting benefits from the use of the cane—this is a process that may take several years).

Most users show a positive interest in using the robotic cane, and even prefer it to a traditional cane. The time required to get adapted and accustomed to the cane is small, around three minutes, proving that the device is very intuitive and simple to use. It provides strong support to the user throughout the movement, even in situations where the surfaces have small irregularities, commonly found outside of domestic environments, while offering a lightweight and compact design that is well adapted to cluttered locations, as is common indoors.

The simplicity of the unicycle kinematics and inverted pendulum dynamics allowed a straightforward control strategy which matched well the typical walking patterns of the users. User perception is that of a very predictable behaviour, thus reducing the adaptation time and increasing the perception of safety.

The implementation of different control methods has shown that the support needs of the users are sufficiently different from each other to further justify the creation of adaptation strategies that can supervise the low level control. The use of the Gini index is part of an experimental evaluation of diversity/inequality indexes for control purposes. This is a novel pathway aiming at, in the future, match specific pathologies with one or more indexes, and enabling its use to select controllers adapted to users. The results reported in the article, with the tuning, in real-time, of the controller gains *via* the Gini index, showed benefits with both cane control methods, enabling a better adjustment to the user’s movement (and hence contributing to user comfort). This however, requires further work and comparative studies with other inequality indexes, *e.g.*, *pq*-means and the Hoyer index.

## Supplemental Information

10.7717/peerj-cs.1563/supp-1 Supplemental Information 1 Data from trial of first user with robotic cane System signals during the test of first elder user with robotic cane

10.7717/peerj-cs.1563/supp-2 Supplemental Information 2 Data from trial of first user with traditional cane System signals during the test of first elder user with traditional cane

10.7717/peerj-cs.1563/supp-3 Supplemental Information 3 Data from trial of second user with robotic cane (part 1) System signals during the first part of the test of second elder user with robotic cane

10.7717/peerj-cs.1563/supp-4 Supplemental Information 4 Data from trial of second user with robotic cane (part 2) System signals during the second part of the test of second elder user with robotic cane

10.7717/peerj-cs.1563/supp-5 Supplemental Information 5 Data from trial of elder user 1 with first robotic cane System signals during the test of elder user 1 with first robotic cane

10.7717/peerj-cs.1563/supp-6 Supplemental Information 6 Data from trial of elder user 2 with first robotic cane System signals during the test of elder user 2 with first robotic cane

10.7717/peerj-cs.1563/supp-7 Supplemental Information 7 Data from trial of elder user 3 with first robotic cane System signals during the test of elder user 3 with first robotic cane

10.7717/peerj-cs.1563/supp-8 Supplemental Information 8 Data from trial of elder user 4 with first robotic cane System signals during the test of elder user 4 with first robotic cane

10.7717/peerj-cs.1563/supp-9 Supplemental Information 9 Data from trial of elder user 5 with first robotic cane System signals during the test of elder user 5 with first robotic cane

10.7717/peerj-cs.1563/supp-10 Supplemental Information 10 Data from trial of elder user 6 with first robotic cane System signals during the test of elder user 6 with first robotic cane

10.7717/peerj-cs.1563/supp-11 Supplemental Information 11 Data from trial of elder user 7 with first robotic cane System signals during the test of elder user 7 with first robotic cane

10.7717/peerj-cs.1563/supp-12 Supplemental Information 12 Data from trial of elder user 1 with second robotic cane System signals during the test of elder user 1 with second robotic cane

10.7717/peerj-cs.1563/supp-13 Supplemental Information 13 Data from trial of elder user 2 with second robotic cane System signals during the test of elder user 2 with second robotic cane

10.7717/peerj-cs.1563/supp-14 Supplemental Information 14 Data from trial of elder user 3 with second robotic cane System signals during the test of elder user 3 with second robotic cane

10.7717/peerj-cs.1563/supp-15 Supplemental Information 15 Data from trial of elder user 4 with second robotic cane System signals during the test of elder user 4 with second robotic cane

10.7717/peerj-cs.1563/supp-16 Supplemental Information 16 Data from trial of elder user 5 with second robotic cane System signals during the test of elder user 5 with second robotic cane

10.7717/peerj-cs.1563/supp-17 Supplemental Information 17 Data from trial of elder user 6 with second robotic cane System signals during the test of elder user 6 with second robotic cane

10.7717/peerj-cs.1563/supp-18 Supplemental Information 18 Data from trial of elder user 7 with second robotic cane System signals during the test of elder user 7 with second robotic cane

10.7717/peerj-cs.1563/supp-19 Supplemental Information 19 Data from trial of normal user 1 with first robotic cane System signals during the test of normal user 1 with first robotic cane

10.7717/peerj-cs.1563/supp-20 Supplemental Information 20 Data from trial of normal user 1 with second robotic cane System signals during the test of normal user 1 with second robotic cane

10.7717/peerj-cs.1563/supp-21 Supplemental Information 21 Data from trial of normal user 2 with first robotic cane System signals during the test of normal user 2 with first robotic cane

10.7717/peerj-cs.1563/supp-22 Supplemental Information 22 Data from trial of normal user 2 with second robotic cane System signals during the test of normal user 2 with second robotic cane

10.7717/peerj-cs.1563/supp-23 Supplemental Information 23 Data from trial of first robotic cane with gini implemented System signals during the test of first robotic cane with gini implemented

10.7717/peerj-cs.1563/supp-24 Supplemental Information 24 Data from trial of first robotic cane without gini implemented System signals during the test of first robotic cane without gini implemented

10.7717/peerj-cs.1563/supp-25 Supplemental Information 25 Data from trial of second robotic cane with gini implemented System signals during the test of second robotic cane with gini implemented

10.7717/peerj-cs.1563/supp-26 Supplemental Information 26 Data from trial of second robotic cane without gini implemented System signals during the test of second robotic cane without gini implemented

10.7717/peerj-cs.1563/supp-27 Supplemental Information 27 Code Data aquisition code of the cane (for Arduino)

## Additional Information and Declarations

### Data Availability

The following information was supplied regarding data availability: Raw measurements and signals of the device during the trials with the users are available in the Supplemental Files.
